# Postoperative hormonal treatment for prevention of endometrioma recurrence after ovarian cystectomy: a systematic review and network meta‐analysis

**DOI:** 10.1111/1471-0528.16366

**Published:** 2020-07-14

**Authors:** R Wattanayingcharoenchai, S Rattanasiri, C Charakorn, J Attia, A Thakkinstian

**Affiliations:** ^1^ Department of Clinical Epidemiology and Biostatistics Faculty of Medicine Ramathibodi Hospital Mahidol University Bangkok Thailand; ^2^ Department of Obstetrics and Gynaecology Faculty of Medicine Ramathibodi Hospital Mahidol University Bangkok Thailand; ^3^ Centre for Clinical Epidemiology and Biostatistics School of Medicine and Public Health Faculty of Health and Medicine University of Newcastle New Lambton Australia

**Keywords:** Endometrioma, hormonal treatment, network meta‐analysis, ovarian cystectomy, recurrence

## Abstract

**Background:**

The efficacy of hormonal regimens for the prevention of endometrioma recurrence in women who have undergone conservative surgery is still controversial.

**Objective:**

To compare the efficacy of different hormonal regimens in this context and to rank them.

**Search strategy:**

MEDLINE and Scopus databases were searched through January 2020.

**Selection criteria:**

Randomised controlled trials (RCTs) or cohorts, comparing the effect of any pair of interventions (i.e. cyclic oral contraceptives [OC], continuous OC, gonadotropin‐releasing hormone agonist [GnRHa], dienogest [DNG], levonorgestrel‐releasing intrauterine system [LNG‐IUS] and expectant management) on endometrioma recurrence were selected.

**Data collection and analysis:**

Data were independently extracted by two reviewers. Relative treatment effects were estimated using network meta‐analysis (NMA) and ranked in descending order.

**Main results:**

Six RCTs (675 patients) and 16 cohorts (3089 patients) were included. NMA of the RCTs involving expectant management, cyclic OC, continuous OC, GnRHa and GnRHa + LNG‐IUS, showed that all hormonal regimens had a nonsignificant lower risk of endometrioma recurrence compared with expectant management. NMA of the cohorts involving expectant, cyclic OC, continuous OC, GnRHa, DNG, LNG‐IUS, GnRHa + OC, and GnRHa + LNG‐IUS indicated that LNG‐IUS, DNG, continuous OC, GnRHa + OC and cyclic OC had a significantly lower risk of endometrioma recurrence than expectant management. LNG‐IUS was ranked highest, followed by DNG and GnRHa + LNG‐IUS. Long‐term use of hormonal treatment either OC or progestin had a significantly lower risk of endometrioma recurrence than expectant treatment.

**Conclusion:**

In the NMA of RCTs, there was no evidence supporting hormonal treatment for postoperative prevention of endometrioma recurrence. This was at odds with the cohort evidence, which found the protective effect of OC and progestin regimens, especially long‐term treatment. Large‐scale RCTs of these agents are still required.

**Tweetable abstract:**

Hormonal regimens given as long‐term treatment tend to reduce risk of endometrioma recurrence after conservative surgery.

## Tweetable abstract

Hormonal regimens given as long‐term treatment tend to reduce risk of endometrioma recurrence after conservative surgery.

## Introduction

Ovarian endometrioma is a major subtype of endometriosis, found up to 55% of women with endometriosis.^1^ Symptoms include dysmenorrhoea, dyspareunia, infertility or pelvic fullness/mass, resulting in adverse effects (AEs) on fertility, personal relationships and quality of life.^2^, ^3^, ^4^

Ovarian cystectomy is one of the conservative surgical procedures for endometrioma and results in decreased ovarian reserve, especially after re‐operating for recurrent disease.^5^, ^6^ Endometrioma recurrence rates range between 30 and 50%,^7^, ^8^ due to the regrowth of residual lesions or de novo lesion formation.^9^ Therefore, postoperative hormonal regimens that suppress ovarian function or reduce menstrual flow may play a role in the prevention of disease recurrence.^10^

Combined oral contraceptives (OC) are the regimens most commonly prescribed for prevention of endometrioma recurrence in clinical practice, and have been recommended as the first‐line treatments in clinical practice guidelines.^11^, ^12^, ^13^ In addition, other types of hormonal treatments (e.g. gonadotropin releasing hormone agonist [GnRHa], depot progestin, dienogest [DNG] or levonorgestrel intrauterine system [LNG‐IUS]) are alternative options for women who are sensitive, or have contraindications, to OC.

The efficacy of these treatments in prevention of endometrioma recurrence has been assessed by two pairwise meta‐analyses.^14^, ^15^ The first meta‐analysis (MA), conducted in 2013,^14^ combined evidence from one randomized controlled trial [RCT] and three cohorts, which indicated the benefit of long‐term (>12 months) use of either cyclic or continuous OCs compared with expectant management. The second meta‐analysis, conducted in 2016,^15^ pooled evidence from three RCTs and one cohort, indicating no difference in endometrioma recurrence but a significantly lower recurrence of dysmenorrhoea in continuous OC users, compared with cyclic regimens given for at least 6 months postoperation. Although these MAs showed a possible benefit of OCs in the prevention of endometrioma recurrence, evidence was based on small numbers and was not robust. In addition, other hormonal regimens such as DNG, LNG‐IUS and GnRHa were not considered in the previous meta‐analyses. Given the increased concern about long‐term use of various hormonal regimens, the harm–benefit ratios of different OC regimens for prevention of endometrioma recurrence are uncertain. Therefore, the present systematic review and network meta‐analysis (NMA) was conducted to estimate the treatment efficacy and safety of the different hormonal regimens used in the prevention of endometrioma recurrence.

## Methods

This systematic review and NMA was conducted according to the preferred reporting items for systematic reviews and meta‐analyses (PRISMA), extension for network meta‐analyses.^16^ The review protocol was registered in PROSPERO (CRD42018105271).

### Search strategy

A literature search was performed using MEDLINE and Scopus databases for identification of relevant articles published from inception to 31 January 2020. Search terms and search strategies were constructed based on population (P), intervention (I), comparator (C) and outcomes (O) (Appendix S1). Identified studies were selected by RW and SR based on information from the title and abstract according to selection criteria. Disagreements were resolved through discussion.

### Study selection and criteria

All RCTs and cohorts conducted in humans were included if they met all the following criteria:

- studied in patients with ovarian endometrioma who underwent ovarian cystectomy;
- compared any pair of the following interventions regardless of dosage, duration of treatment and drug discontinuation: OC, DNG, LNG‐IUS, GnRHa and expectant treatment;
- had any of the following outcomes: endometrioma recurrence, dysmenorrhoea/pelvic pain recurrence or adverse hormonal effects.

Studies were excluded if they provided insufficient data for analysis after three attempts to contact the author.

The primary outcome was endometrioma recurrence, defined as ultrasound identification of a round mass with diffuse homogeneous ground‐glass echoes, in an individual who had undergone ovarian cystectomy.^17^ The secondary outcome was dysmenorrhoea/pelvic pain recurrence, defined as presence of menstrual pain or pain in the pelvic area occurring any time after postoperative pain relief.^18^ Adverse hormonal effects including metrorrhagia and amenorrhoea were also considered.

### Data extraction

Two reviewers (RW and SR) independently extracted data using a standardised data extraction form; this captured age, revised American Society for Reproductive Medicine (rASRM) score, rASRM staging, characteristics of endometrioma (i.e. size of cyst, bilateral cyst), presence of pelvic adhesion, duration of treatment and follow up. Type of interventions and outcomes of interest along with definitions reported in each study were extracted. Frequency data or summary statistics with standard errors were also extracted for data pooling. Disagreements were discussed and resolved by a third reviewer (AT).

### Risk of bias assessment

Two reviewers (RW and SR) independently assessed the quality of the studies. The Cochrane collaboration tool for assessing risk‐of‐bias version 5.1.0^19^ was used for assessment of RCTs in six domains, including selection bias, performance bias, attrition bias, detection bias, reporting bias and other sources of bias. Each item was classified as low, high or unclear risk of bias.

Cohort studies were assessed using the risk of bias in non‐randomised studies of interventions (ROBINS‐I) assessment tool,^20^ which assessed seven domains: confounding, selection of participants, classification of interventions, deviations from intended interventions, missing data, measurement of outcomes, and selection of the reported result. Each domain was classified as low, moderate, serious, critical risk of bias or no information. Disagreement was resolved by consensus after discussion between both reviewers.

### Statistical analysis

#### Direct meta‐analysis (DMA)

RCTs and cohort studies were analysed separately. A risk ratio (RR) along with its 95% confidence interval (CI) of each study was estimated from frequency data. In the studies with zero events, a continuity correction was performed by adding 0.5 to all cells, allowing estimation of RR. The RRs were pooled across studies using a fixed‐effect model by an inverse‐variance method if there was no heterogeneity, otherwise a random‐effect model using the DerSimonian and Laird method was applied. Heterogeneity was assessed by Cochrane's Q test and Higgins's *I* ^2^ statistic.^21^ Publication bias was assessed using funnel plots, Egger's tests^22^ and contour‐enhanced funnel plots.^23^

#### Network meta‐analysis

Relative treatment effects between hormonal regimens were compared using an NMA framework. Treatment regimens were numerically coded for expectant management (1), cyclic OC (2), continuous OC (3), GnRHa (4), DNG (5), LNG‐IUS (6), GnRHa + OC (7) and GnRHa + LNG‐IUS (8). A two‐stage approach using multivariate random effects meta‐analysis with consistency model was applied to compare treatment efficacy across the network.^24^ Multiple treatment comparisons were then estimated.

The inconsistency assumption was checked using an adjusted design‐by‐treatment interaction model.^25^ If there was evidence of inconsistency (*P*‐value for global test < 0.05), a loop‐specific approach was used to identify the treatment arms and studies which contributed most to the inconsistency. The probabilities of being the best treatment (lowest RR for recurrence of endometrioma, dysmenorrhoea/pelvic pain and AEs) were estimated and ranked using a rankogram and the surface under the cumulative ranking curve (SUCRA) method.^26^ Predictive intervals were estimated and plotted by considering heterogeneity within and between treatment comparisons. Subgroup analysis by treatment continuity up to follow‐up time was performed. Publication bias for NMA was assessed using comparison‐adjusted funnel plots.^27^ If this was present, sensitivity analysis was performed by excluding the studies with low precision (i.e. standard error of effect size larger than 75 percentiles) to see robustness of results.

All analyses were performed using STATA software package, version 15.0 (Stata Corp, College Station, TX, USA). A two‐sided *P*‐value of <0.05 was set as the threshold for statistical significance, except for the test of heterogeneity, in which a *P*‐value <0.10 was used.

### Patient involvement

There was no patient involvement because this meta‐analysis used data from published studies.

## Results

**Figure 1:**
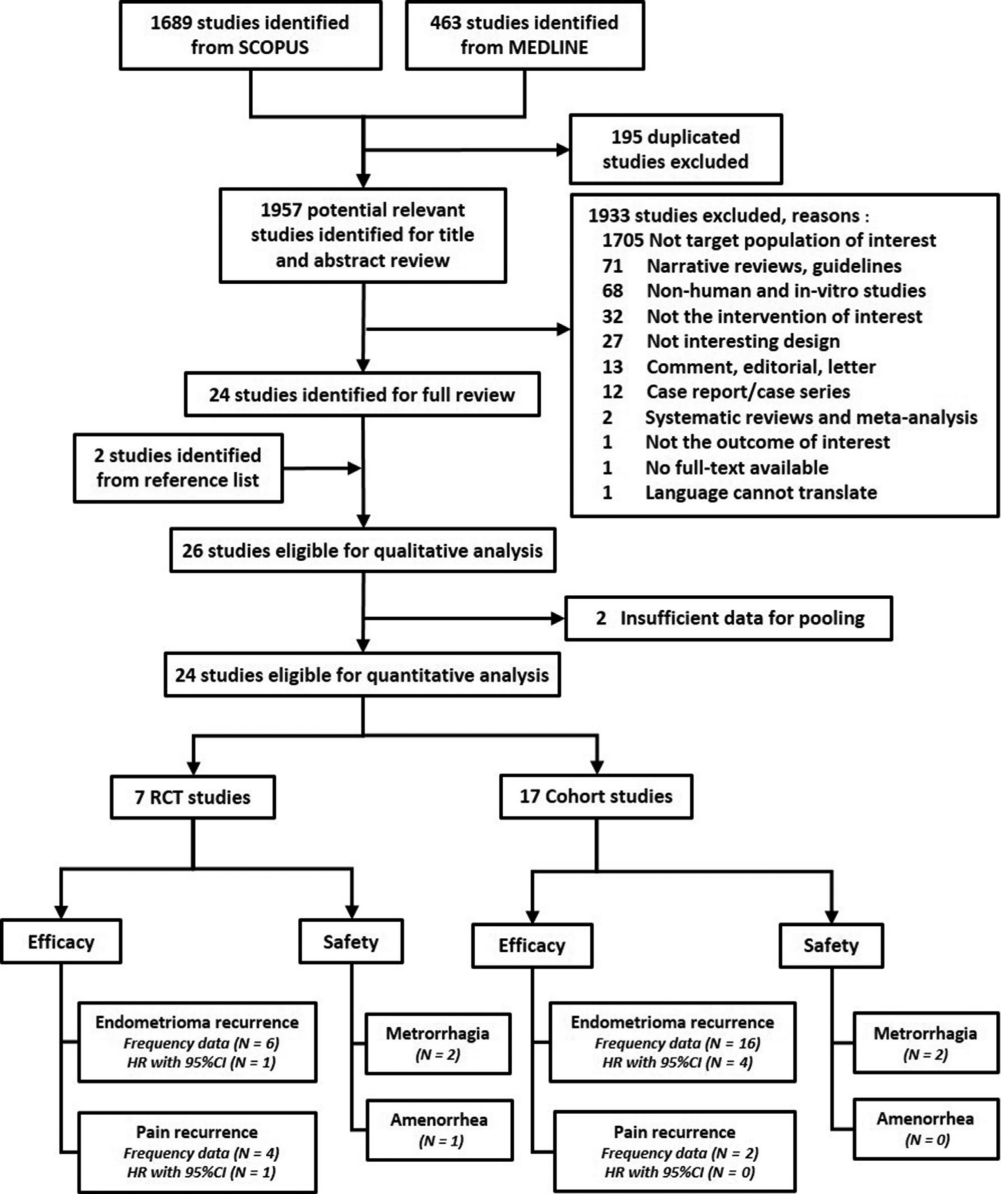
Flow diagram of study selection.

A total of 2152 studies were identified from PubMed and Scopus. After deleting duplicates, 24 plus two additional studies identified from reference lists met the inclusion criteria (i.e. eight RCTs and 18 cohorts) and were included for qualitative analysis. After two studies (i.e. RCT^28^ and cohort^29^) were excluded due to insufficient data for pooling, 24 studies remained (i.e. seven RCTs and 17 cohorts) for the quantitative analysis (Figure 1).

### Characteristics of included studies

Among the eight RCTs (*n* = 1116),^28^, ^30^, ^31^, ^32^, ^33^, ^34^, ^35^, ^36^ mean age ranged from 27.5 to 33.9 years. Most studies (62.5%) enrolled patients with moderate‐to‐severe disease. Duration of postoperative treatments was 3–30 with a median 6 months, whereas follow‐up time was 18–60 with a median of 24 months. Only three studies^33^, ^34^, ^36^ continued treatment up to the end of follow up; in those studies, continuous OC versus cyclic OC were continued up to 24 months^33^, ^34^ and LNG‐IUS was continued up to 30 months^36^ (Table 1). Six,^30^, ^31^, ^32^, ^33^, ^35^, ^36^ four,^30^, ^34^, ^35^, ^36^ two^34^, ^36^ and one^36^ RCT, respectively, reported endometrioma recurrence, dysmenorrhoea/pelvic pain recurrence, AEs and time to event outcome (Figure 1).

**Table 1:** Characteristics of included RCTs

Author, Year	Country	Included participants	Mean age (yr)	Interventions	Duration of treatment (mo)	*N*	Definition of outcomes		Follow up (mo)
							Endometrioma recurrence	Pain recurrence	
Yang,^28^ 2019	China	Endometriosis stage 3 & 4	28.7	1. GnRHa 2. Expectant management	6 –	65 65	Not defined	Not defined	24
Chen,^36^ 2017	Taiwan	Symptomatic endometrioma stage 3 & 4	33.9	1. GnRHa plus LNG‐IUS 2. GnRHa	Entire FU 6	40 40	Cyst ≥ 3 cm	VAS score >50 mm	30
Muzii,^35^ 2011	Italy	Symptomatic endometrioma > 3 cm	30.5	1. Continuous OC 2. Cyclic OC	6 6	29 28	Cyst > 3 cm	VAS score > grade 4	24
Seracchioli,^34^ 2010	Italy	Symptomatic endometrioma stage 3 & 4	29.5	1. Continuous OC 2. Cyclic OC 3. Expectant management	24 24 –	104 103 104	Not assessed	VAS score ≥ grade 4	24
Seracchioli,^33^ 2010	Italy	Endometrioma > 4 cm; stage 3 & 4	29.5	1. Continuous OC 2. Cyclic OC 3. Expectant management	24 24 –	79 81 79	Cyst ≥ 1.5 cm	Not assessed	24
Sesti,^32^ 2009	Italy	Symptomatic endometrioma	30.8	1. GnRHa 2. Continuous OC 3. Expectant management	6 6 –	65 64 65	Cyst > 2 cm	Not assessed	18
Loverro,^31^ 2008	Italy	Symptomatic endometriosis stage 3 & 4 (endometrioma‐subgroup)	N.A	1. GnRHa 2. Expectant management	3 –	19 16	Not defined	Not assessed	60
Muzii,^30^ 2000	Italy	Symptomatic endometrioma	27.5	1. Cyclic OC 2. Expectant management	6 –	35 35	Not defined	VAS score ≥ grade 4	36

N.A.: not available.

Among the 18 cohorts^18^, ^29^, ^37^, ^38^, ^39^, ^40^, ^41^, ^42^, ^43^, ^44^, ^45^, ^46^, ^47^, ^48^, ^49^, ^50^, ^51^, ^52^ (*n* = 3316), mean age ranged from 27.3 to 37.9 years. Most studies (61.1%) enrolled patients with moderate‐to‐severe disease. Duration of treatments was 3–60 with a median of 24 months, whereas duration of follow up was 6–60 with a median of 24 months. Ten studies^37^, ^38^, ^39^, ^40^, ^41^, ^44^, ^45^, ^46^, ^51^, ^52^ had continued hormonal treatments up to follow‐up time (Table S1). Sixteen,^18^, ^37^, ^38^, ^40^, ^41^, ^42^, ^43^, ^44^, ^45^, ^46^, ^47^, ^48^, ^49^, ^50^, ^51^, ^52^ two,^39^, ^44^ two^39^, ^44^ and four^38^, ^40^, ^45^, ^49^ cohorts, respectively, reported endometrioma recurrence, dysmenorrhoea/pelvic pain recurrence, AEs and time to event outcome (Figure 1). Therefore, quantitative analysis was focused only on endometrioma recurrence.

### Risk of bias assessment

For RCTs, all studies had low risk for selective outcome reporting. Seven studies (87.5%) had low risk for random sequence generation. Allocation concealment was appropriately performed in five trials (62.5%). All studies had low risk for blinding of participants for both endometrioma and pain recurrence outcomes. For detection bias domain, all studies had low risk for blinding of endometrioma recurrence assessment, whereas all had high risk for blinding of pain recurrence assessment. Five studies (62.5%) had incomplete outcome data. Seven studies (87.5%) were potentially biased from applying per protocol analysis or allowing cross‐over of subjects (Figure S1).

Among cohorts, five studies (29.4%) had a critical risk of confounding domain. Two studies (11.7%) had serious risk for selection of participants. All studies had low risk in classification of interventions. Eight (47.1%) and four (23.5%) studies had moderate risk for deviations from intended intervention and missing data domain, respectively. Two studies (11.7%) had serious risk in measurement of outcomes. For the overall risk of bias, five (27.8%), eight (44.4%) and five (27.8%) studies had critical, serious and moderate risk of bias, respectively (Table S2).

### RCTs

DMA was performed for cyclic OC versus expectant management^30^, ^33^, continuous OC versus expectant management^32^, ^33^, GnRHa versus expectant management^31^, ^32^ and continuous OC versus cyclic OC^33^, ^35^ on endometrioma recurrence. Cyclic OC, continuous OC and GnRHa respectively had a 41% (RR 0.59, 95% CI 0.31–1.12), 47% [RR 0.53, 95% CI 0.18–1.57) and 21% (RR 0.79, 95% CI 0.35–1.79) lower risk of endometrioma recurrence than expectant treatment, with an *I* ^2^ of 10, 70.1 and 18.3%, respectively. Continuous OC had a 47% (RR 0.53, 95% CI 0.22–1.32) lower risk of endometrioma recurrence compared with cyclic OC, with an *I* ^2^ of 0%. None of these risks was statistically significant (Figure S2).

Six RCTs^30^, ^31^, ^32^, ^33^, ^35^, ^36^ (*n* = 675) were pooled in the NMA. A network map was constructed consisting five regimens (i.e. expectant management, cyclic OC, continuous OC, GnRHa and GnRHa + LNG‐IUS) (Figure S3). The most informative direct comparison contributing to the network was GnRHa + LNG‐IUS versus GnRHa (21.4%), followed by GnRHa versus expectant management (19.5%) and GnRHa versus continuous OC (18.4%) (Figure S4).

The NMA indicated that GnRHa + LNG‐IUS was the most effective regimen when compared with expectant management, with a pooled RR of 0.48 (95% CI 0.08–2.73), followed by continuous OC, GnRHa and cyclic OC with corresponding pooled RRs of 0.59 (95% CI 0.23–1.54), 0.72 (95% CI 0.23–2.26) and 0.90 (95% CI 0.28–2.92), respectively; none of these was statistically significant. Among hormonal regimens, GnRHa + LNG‐IUS seemed to be superior to GnRHa monotherapy, continuous OC and cyclic OC, with pooled RRs of 0.67 (95% CI 0.18–2.48), 0.81 (95% CI 0.13–4.97) and 0.53 (95% CI 0.07–3.94), respectively. The ranking generated by SUCRA indicated that GnRHa + LNG‐IUS ranked first (SUCRA 72.2), followed by continuous OC (SUCRA 64.9) and GnRHa (SUCRA 49.6) (Table 2, Figure S5). The global inconsistency test showed no evidence of inconsistency (Chi‐square test = 6.55, df = 4, *P* = 0.162).

**Table 2:** Multiple treatment comparison of RCT network for endometrioma recurrence outcome

Reference treatment	Risk ratio (95% CI)				
	Expectant management	Cyclic OC	Continuous OC	GnRHa	GnRHa + LNG‐IUS
Expectant management	**26.7**	0.90 (0.28–2.92)	0.59 (0.23–1.54)	0.72 (0.23–2.26)	0.48 (0.08–2.73)
Cyclic OC	1.11 (0.34–3.59)	**36.5**	0.66 (0.19–2.25)	0.80 (0.18–3.62)	0.53 (0.07–3.94)
Continuous OC	1.69 (0.65–4.39)	1.52 (0.44–5.21)	**64.9**	1.21 (0.34–4.26)	0.81 (0.13–4.97)
GnRHa	1.39 (0.44–4.37)	1.26 (0.28–5.70)	0.83 (0.23–2.90)	**49.6**	0.67 (0.18–2.48)
GnRHa + LNG‐IUS	2.09 (0.37–11.90)	1.88 (0.25–13.96)	1.24 (0.20–7.62)	1.50 (0.40–5.57)	**72.2**

Each off‐diagonal cell contains RR (95% CI). Each diagonal cell contains SUCRA.

Bold indicates the values of sucra.

A sensitivity analysis was performed by excluding two studies^33^, ^36^ with continued treatments of continuous OC,^33^ cyclic OC^33^ and LNG‐IUS^36^ until time at endometrioma assessment at 24–30 months. The pooled RRs for GnRHa, continuous OC and cyclic OC versus expectant management were 0.79 (95% CI 0.35–1.77), 0.99 (95% CI 0.45–2.20) and 2.33 (95% CI 0.35–15.74), respectively (Table S3). This could be interpreted as short‐term effects of GnRH (i.e. 3–6 months) being to be more effective than continuous OC or cyclic OC (i.e. 6 months).

Transitivity was further assessed by exploring characteristics for five pairwise comparisons (i.e. cyclic OC versus expectant management, continuous OC versus expectant management, GnRHa versus expectant management, continuous OC versus cyclic OC and GnRHa versus continuous OC) across studies. The results indicated that percent rASRM stage, bilateral cyst, duration of treatment and duration of follow‐up were quite different across studies, whereas the rest did not show much difference (Table S4). A comparison‐adjusted funnel plot showed no asymmetry, reflecting the absence of any association between study size and study effect (Figure S6).

### Cohorts

DMA was performed and showed that cyclic OC and DNG had respectively about a 64% (RR 0.36, 95% CI 0.18–0.72; *I* ^2^ = 65.5%) and an 86% (RR 0.14, 95% CI 0.06–0.33; *I* ^2^ = 0%) significantly lower risk of endometrioma recurrence compared with expectant management. GnRHa + OC also had a 68% (RR 0.32, 95% CI 0.15–0.71; *I* ^2^ = 28.2%) significantly lower risk of endometrioma recurrence than GnRHa, but this decrease was not significant compared with expectant management (RR 0.72, 95% CI 0.06–8.21; *I* ^2^ = 87.1%). Conversely, GnRHa had an 11% (RR 1.11, 95% CI 0.67–1.83; *I* ^2^ = 62.2% higher endometrioma recurrence when compared with expectant management, but this increase was not significant (Figure S7).

Sources of heterogeneity for comparisons of cyclic OC versus expectant management and GnRHa versus expectant management were explored across studies. The results indicated that none of the baseline characteristics was a source of heterogeneity (Table S5). The results of Egger's test and funnel plot showed no evidence of publication bias for either pooling (Figure S8).

Data from the 16 cohorts^18^, ^37^, ^38^, ^40^, ^41^, ^42^, ^43^, ^44^, ^45^, ^46^, ^47^, ^48^, ^49^, ^50^, ^51^, ^52^ (*n* = 3089) were pooled in NMA of eight regimens (i.e. expectant management, cyclic OC, continuous OC, GnRHa, DNG, LNG‐IUS, GnRHa + OC and GnRHa + LNG‐IUS) (Figure S9). Cyclic OC versus expectant management (27.0%), GnRHa versus expectant management (15.5%) and DNG versus expectant management (10.7%) were the main contributors (Figure S10).

Pooled relative treatment effects demonstrated that LNG‐IUS, DNG, continuous OC, GnRHa + OC and cyclic OC had a significantly lower endometrioma recurrence compared with expectant management, with pooled RRs of 0.05 (95% CI 0.00–0.98), 0.14 (95% CI 0.05–0.43), 0.30 (95% CI 0.11–0.77), 0.33 (95% CI 0.15–0.71), and 0.35 (95% CI 0.20–0.60), respectively. LNG‐IUS and DNG seemed to be better than other active regimens in lowering endometrioma recurrence, but their effects were significant only when compared with GnRHa, with a pooled RR of 0.05 (95% CI 0.00–0.95) and 0.14 (95% CI 0.04–0.46), respectively. Continuous and cyclic OCs had a significantly lower risk of endometrioma recurrence than GnRHa alone with pooled RRs of 0.29 (95% CI 0.11–0.79) and 0.34 (95% CI 0.17–0.67), respectively. Addition of GnRHa to either OC regimen or LNG‐IUS did not significantly alter the treatment effect (Table 3).

**Table 3:** Multiple treatment comparison of cohort network for endometrioma recurrence outcome

Reference treatment	Risk ratio (95% CI)							
	Expectant management	Cyclic OC	Continuous OC	GnRHa	DNG	LNG‐IUS	GnRHa + OC	GnRHa + LNG‐IUS
Expectant management	**8.7**	0.35 (0.20–0.60)	0.30 (0.11–0.77)	1.03 (0.65–1.62)	0.14 (0.05–0.43)	0.05 (0.00–0.98)	0.33 (0.15–0.71)	0.15 (0.02–1.04)
Cyclic OC	2.86 (1.66, 4.91)	**45.9**	0.85 (0.33–2.19)	2.93 (1.49–5.79)	0.40 (0.12–1.36)	0.15 (0.01–2.91)	0.95 (0.38–2.33)	0.43 (0.06–3.15)
Continuous OC	3.37 (1.30, 8.70)	1.18 (0.46–3.04)	**54.2**	3.46 (1.26–9.49)	0.47 (0.11–2.02)	0.17 (0.01–3.77)	1.11 (0.34–3.63)	0.50 (0.06–4.27)
GnRHa	0.97 (0.62, 1.53)	0.34 (0.17–0.67)	0.29 (0.11–0.79)	**7.3**	0.14 (0.04–0.46)	0.05 (0.00–0.95)	0.32 (0.15–0.69)	0.15 (0.02–1.01)
DNG	7.16 (2.33, 22.01)	2.51 (0.74–8.53)	2.13 (0.49–9.14)	7.36 (2.19–24.67)	**77.3**	0.37 (0.02–8.60)	2.37 (0.61–9.15)	1.07 (0.11–10.02)
‐IUS	19.55 (1.02, 374.49)	6.84 (0.34–136.21)	5.80 (0.26–127.14)	20.07 (1.05–383.44)	2.73 (0.12–64.02)	**87.3**	6.47 (0.37–111.99)	2.93 (0.32–27.16)
GnRHa + OC	3.02 (1.41, 6.50)	1.06 (0.43–2.61)	0.90 (0.28–2.93)	3.10 (1.46–6.60)	0.42 (0.11–1.63)	0.15 (0.01–2.68)	**48.7**	0.45 (0.08–2.68)
GnRHa + LNG‐IUS	6.68 (0.96, 46.38)	2.34 (0.32–17.21)	1.98 (0.23–16.80)	6.86 (0.99–47.42)	0.93 (0.10–8.71)	0.34 (0.04–3.17)	2.21 (0.37–13.11)	**70.6**

Each off‐diagonal cell contains RR (95% CI). Each diagonal cell contains SUCRA.

Bold indicates the values of sucra.

The probability of being the best treatment in lowering recurrence was highest for LNG‐IUS (SUCRA 87.3), followed by DNG (SUCRA 77.3) and GnRHa + LNG‐IUS (SUCRA 70.6) (Table 3, Figure S11). The global inconsistency test showed no evidence of inconsistency (Chi‐square = 12.02, df = 8, *P* = 0.150). Transitivity was also explored, which indicated that the percentage of rASRM stage IV, rASRM score, bilateral cyst, duration of treatment and follow up were quite different across studies (Table S6). A comparison‐adjusted funnel plot of NMA showed no asymmetry (Figure S12).

Subgroup analysis was performed by continuity of treatments. Ten cohorts^37^, ^38^, ^40^, ^41^, ^44^, ^45^, ^46^, ^47^, ^51^, ^52^ (*n* = 1997) continued hormonal treatments up to end of a follow up of 12–60 months. The relative treatment effects of LNG‐IUS, DNG, GnRHa + OC, continuous OC and cyclic OC did not change much from overall pooling, with the RRs of 0.04 (95% CI 0.00–0.61), 0.14 (95% CI 0.05–0.37), 0.23 (95% CI 0.09–0.61), 0.25 (95% CI 0.11–0.56) and 0.30 (95% CI 0.18–0.48) (Table S7). In addition, excluding the two studies^40^, ^45^ with LNG‐IUS did not greatly change the effects of DNG, GnRHa + OC, continuous OC or cyclic OC (Table S8).

Five cohorts^18^, ^42^, ^43^, ^48^, ^50^ (*n* = 721) used hormonal treatments for 3–12 months, but discontinued before the follow up. Relative treatment of three regimens (cyclic OC, GnRHa and GnRHa + OC) was worse for endometrioma recurrence than was expectant management (Table S9).

## Discussion

### Main findings

We conducted a systematic review and NMA of RCTs and cohorts comparing endometrioma recurrence among various hormonal regimens and expectant management. The pooled RR point estimates from the RCT‐NMA indicated that all hormonal regimens could lower endometrioma recurrence compared with expectant management, but none reached statistical significance. Pooled relative treatment effects from cohort NMA found a significantly lower endometrioma recurrence risk in LNG‐IUS, DNG, continuous OC, GnRHa + OC and cyclic OC compared with expectant management. Summarising the evidence from RCT NMA, GnRHa + LNG‐IUS was the most effective regimen followed by continuous OC and GnRHa. Evidence from the cohort NMA suggested that LNG‐IUS ranked first in lowering endometrioma recurrence, followed by DNG and GnRHa + LNG‐IUS.

None of the hormonal regimens given as a short‐term treatment for about 3–6 months lowered endometrioma recurrence compared with expectant management. However, long‐term or continuous use of any hormonal regimen inhibiting ovulation could significantly lower endometrioma recurrence compared with expectant management in which DNG was the most effective regimen, followed by GnRHa + OC.

Relative treatment effects estimated by RCT and cohort NMAs were inconsistent in two comparisons: GnRHa versus expectant management and GnRHa versus cyclic OC (Table S10). Among these comparisons, only GnRHa versus expectant management was directly compared in both RCTs and cohorts, with opposite relative treatment effects. For RCTs, based on reported data, chanracteristics of patients in expectant management and GnRHa groups were quite comparable, except for the percentage of bilateral cyst, which was a bit lower in expectant management than in GnRHa groups. For cohorts, cyst size and percentage of bilateral cyst were higher in GnRHa than in expectant management groups, whereas bilateral cyst and rASRM stage IV were higher in GnRHa than in cyclic OC. This explains why GnRHa decreased the recurrence of endometrioma in RCTs but was a higher risk in cohorts (Table S11). However, this was only considered based on the data available, as not all RCTs/cohorts reported baseline risks (Table S12).

### Strengths and limitations

The strength of our study is that we considered most current regimens for prevention of endometrioma recurrence. Treatments other than OC were added to those from the previous studies;^14^, ^15^ application of the NMA framework allowed multiple relative treatment comparisons and ranked the best agents for preventing endometrioma recurrence given the evidence to date from both RCTs and cohorts.

There were some limitations that we could not avoid. First, there was a limited number of relevant studies and their sample sizes were rather small, especially in the RCT network, which might cause uncertainty of the estimated treatment effect, low power to detect the consistency assumption by global test, and limited generalisability of our findings.

Second, duration of treatment and follow up among regimens varied greatly, which may potentially affect the outcome. However, subgroup analysis by continuity of treatment up to end of follow up was performed to assess effects of continuity of treatments.

Third, other few important outcomes such as endometrioma pain and recurrence that required surgery were not considered due to a small number of studies reporting these outcomes.

Finally, most RCTs were potentially biased because they applied per protocol analysis.

### Interpretation

Our findings from RCT and cohort NMA confirm and extend the previous two meta‐analyses^14^, ^15^ that supported the benefit of OCs in the prevention of endometrioma recurrence. Apart from cyclic and continuous OCs, hormonal regimens involving GnRHa + LNG‐IUS and GnRHa seemed to decrease endometrioma recurrence better than expectant management does, although none of these was statistically significant. However, the duration of GnRHa administration should be no longer than 6 months due to unfavourable effects (e.g. decreased bone mineral density, menopausal symptoms, etc.). Their effects in the suppression of ovarian function will vanish as soon as the treatment are discontinued. Therefore, continuity and safe long‐term treatments are still required. Large‐scale RCTs considering both efficacy and safety are still required to confirm the effectiveness of GnRHa + LNG‐IUS before applying these results in clinical practice.

The evidence from the cohort NMA found that LNG‐IUS and DNG were the top two ranked treatments, lowering the risk of endometrioma by 95 and 86%, compared with expectant management, respectively. Although the estimated treatment effects were significant, the results were derived from few studies. Moreover, cohort designs are more susceptible to selection bias. To confirm the protective effect of these regimens, more high‐quality studies are needed. Combining RCT and cohort data using a hierarchical mixed‐effect logit model and adjusting for study design,^53^ suggested that DNG was still better than expectant management and other hormonal regimens (Table S13).

Our findings complied with clinical practice guidelines^11^, ^12^, ^13^ that only long‐term hormonal treatment can prevent endometrioma recurrence. All hormonal regimens inhibiting ovulation are better than no treatment, therefore cost and AEs of each regimen should be considered in practice. The ovulation inhibition effect of LNG‐IUS is theoretically less than 50% after 3 months of insertion,^54^ which is the main mechanism of endometrioma.^55^, ^56^ However, our findings showed that continuous treatment with LNG‐IUS might be beneficial. Therefore, there may be other possible mechanisms that LNG‐IUS might work through and which would be of benefit in the prevention of endometrioma recurrence.^57^, ^58^

## Conclusion

The best evidence derived from the RCT network suggested that GnRHa plus LNG‐IUS was the most effective regimen in lowering risk of endometrioma recurrence, followed by continuous OC and GnRHa. Nevertheless, this was based on nonsignificant treatment effects, perhaps because of the small number of RCTs. Long‐term use of DNG had a favourable effect in prevention of endometrioma recurrence, but the evidence was derived from a cohort network. Therefore, applying these treatments for prevention of endometrioma recurrence should be considered carefully for individual patients.

### Disclosure of interests

None declared. Completed disclosure of interests forms are available to view online as supporting information.

### Contribution to authorship

RW, SR, CC and AT were involved in the conception and design of the protocol. RW, SR and AT designed the search strategy. RW and SR selected eligible studies, extracted data and assessed the quality of the included studies. Data analysis and interpretation were carried out by RW, SR and AT. Drafts of the manuscript were prepared by RW and SR, and critically revised by JA and AT. All authors contributed to the drafts and final version of the manuscript and approved the final review.

### Details of ethics approval

Not applicable.

### Funding

This study received no funding.

## Supporting information


**Appendix S1.** Search strategies and results MEDLINE via PubMed and Scopus.

**Figure S1.** Risk of bias assessments for RCTs.
**Figure S2.** Forest plot of pairwise meta‐analysis of endometrioma recurrence based on RCT network.
**Figure S3.** Network map of RCTs comparing hormonal treatments for prevention of endometrioma recurrence.
**Figure S4.** Network contribution plot of RCTs for endometrioma recurrence outcome.
**Figure S5.** Rankograms for hormonal network of RCTs showing the probability for each regimen being at a particular order in lowering endometrioma recurrence.
**Figure S6.** Comparison‐adjusted funnel plot for network meta‐analysis of RCTs on endometrioma recurrence outcome.
**Figure S7.** Forest plot of pairwise meta‐analysis of endometrioma recurrence based on cohort network.
**Figure S8.** Funnel plot of cyclic OC versus expectant management and GnRHa versus expectant management for endometrioma recurrence based on cohort data.
**Figure S9.** Network map of cohorts comparing among hormonal treatments for prevention of endometrioma recurrence.
**Figure S10.** Network contribution plot of cohorts for endometrioma recurrence outcome.
**Figure S11.** Rankograms for hormonal network of cohorts showing the probability for each regimen being at a particular order in lowering endometrioma recurrence.
**Figure S12.** Comparison‐adjusted funnel plot for network meta‐analysis of cohorts on endometrioma recurrence outcome.

**Table S1.** Characteristics of included cohorts.
**Table S2.** Risk of bias assessment of included cohort studies.
**Table S3.** Multiple treatment comparison of RCTs network for endometrioma recurrence outcome: a sensitivity analysis by excluding two studies with continued hormonal treatments
**Table S4.** Distribution of effect modifiers between each pairwise comparison across network of RCTs for endometrioma recurrence.
**Table S5.** Explore sources of heterogeneity: meta‐regression
**Table S6.** Distribution of effect modifiers between each pairwise comparison across network of cohorts for endometrioma recurrence.
**Table S7.** Multiple treatment comparison of cohort network for endometrioma recurrence outcome in a subgroup continued hormonal treatment group.
**Table S8.** Multiple treatment comparison of cohort network for endometrioma recurrence outcome in a subgroup continued hormonal treatment group with removing LNG‐IUS.
**Table S9.** Multiple treatment comparison of cohort network for endometrioma recurrence outcome in a subgroup with discontinued treatment up to last follow up.
**Table S10.** Relative effect estimates of treatment comparisons of RCT and cohort network for endometrioma recurrence.
**Table S11.** Characteristics of subjects and RRs (95% CI) in GnRHa versus expectant management comparison; RCT and cohort study.
**Table S12.** Characteristics of subjects in expectant management, cyclic OC and GnRHa intervention among RCTs and cohort studies.
**Table S13.** Estimation of treatment effects on recurrence adjusting for study designs: a multilevel, mixed‐effect logit model.

Supplementary Material

Supplementary Material

Supplementary Material

Supplementary Material

Supplementary Material
