# Supplementary material for: Postoperative hormonal treatment for prevention of endometrioma recurrence after ovarian cystectomy: a systematic review and network meta‐analysis
Source: BJOG. 2020 Jul 14;128(1):25–35. doi: 10.1111/1471-0528.16366 (PMC7754428; doi:10.1111/1471-0528.16366)
Supplement: Supplementary file 1 — Appendix S1. Search strategies and results MEDLINE via PubMed and Scopus. [file BJO-128-25-s003.pdf]

Appendix S1. Search strategies and results.

a) **MEDLINE via PubMed** (Date of last search: 31st January 2020)

Domain(S)	Search number	Query	Items found
Patients	#1	Search endometrioma	27616
P1	#2	Search endometriomas	27528
Disease	#3	Search "endometriotic cyst"	256
	#4	Search "endometriotic cysts"	287
	#5	Search “chocolate cyst"	85
	#6	Search “chocolate cysts"	71
	#7	Search #1 OR #2 OR #3 OR #4 OR #5 OR #6	27837
P2	#8	Search laparosco*	138798
General	#9	Search laparoscopy	109873
Surgery	#10	Search "gynecological surgery"	1943
	#11	Search "gynecologic surgery"	3991
	#12	Search "gynaecological surgery”	1293
	#13	Search "gynaecologic surgery"	172
	#14	Search "minimal invasive surgery"	1180
	#15	Search "minimally invasive surgery"	15114
	#16	Search "minimally invasive gynecologic surgery"	358
	#17	Search #8 OR #9 OR #10 OR #11 OR #12 OR #13 OR #14 OR #15 OR #16	153480
P3	#18	Search "ovarian cystectomy"	465
Specific	#19	Search "ovarian cystectomies"	39
Surgery	#20	Search "ovarian cyst removal"	9
	#21	Search "endometrioma removal"	5
	#22	Search "endometriotic cystectomy"	10
	#23	Search "laparoscopic cystectomy"	315
	#24	Search "laparoscopic ovarian cystectomy"	141
	#25	Search #18 OR #19 OR #20 OR #21 OR #22 OR #23 OR #24	784
P	#26	Search #7 AND #17 OR #25	6690
Interventions	#27	Search hormonal	121598
And	#28	Search "oral contraceptives"	33929
Comparators	#29	Search "oral contraceptive"	11535
	#30	Search "oral contraception"	2631
	#31	Search "combined pills"	125
	#32	Search estroprogestin	67
	#33	Search progestin	77005
	#34	Search "dienogest"	526
	#35	Search "depot medroxyprogesterone acetate"	939
	#36	Search "Depo-provera"	889

	#37	Search "LNG-IUS"	715
	#38	Search Mirena	6134
	#39	Search "gonadotropin releasing hormone agonist"	1821
	#40	Search "gonadotropin releasing hormone agonists"	569
	#41	Search "GnRH agonist"	3628
	#42	Search "GnRH agonists"	1299
	#43	Search "GnRH analogue"	899
	#44	Search expectant	5700
I and C	#45	Search #27 OR #28 OR #29 OR #30 OR #31 OR #32 OR #33 OR #34 OR #35 OR #36 OR #37 OR #38 OR #39 OR #40 OR #41 OR #42 OR #43 OR #44	227034
Outcomes	#46	Search recurr*	683733
recurrences	#47	Search recurrences	491840
	#48	Search #46 OR #47	684065
dysmenorrhea	#49	Search dysmenor*	6895
	#50	Search "painful menstruation"	101
	#51	Search "menstrual pain"	598
	#52	Search #49 OR #50 OR #51	7127
pelvic pain	#53	Search "pelvic pain"	10867
	#54	Search "pelvic pains"	100
	#55	Search #53 OR #54	10922
O	#56	Search #48 OR #52 OR #55	699626
P, I/C & O	#57	Search #26 AND #45 AND #56	463

b) Scopus (Date of last search: 31st January 2020)

**Domain(s)**	**Search number**	**Query**	**Items found**
Patients	#1	ALL (endometrioma)	6819
P1 Disease	#2	ALL (endometriomas)	7857
	#3	ALL (*"ovarian endometriosis"*)	5010
	#4	ALL (*"endometriotic cyst"*)	2444
	#5	ALL (*"chocolate cyst"*)	789
	#6	#1 OR #2 OR #3 OR #4 OR #5	12224
P2	#7	ALL (laparoscopy)	179599
General	#8	ALL ("laparoscopic surgery")	116963
surgery	#9	ALL ("laparoscopic assisted surgery")	2561
	#10	ALL ("gynecological surgery")	14748
	#11	ALL ("gynecologic surgery")	31757
	#12	ALL ("gynaecologic surgery")	718
	#13	ALL ("minimal invasive surgery")	4063
	#14	ALL ("minimally invasive surgery")	75401

	#15	ALL ("minimally invasive gynecologic surgery")	652
	#16	#7 OR #8 OR #9 OR #10 OR #11 OR #12 OR #13 OR #14 OR #15	300404
P3	#17	ALL (*"ovarian cystectomy"*)	1905
Specific	#18	ALL (*"ovarian cystectomies"*)	58
surgery	#19	ALL (*"ovarian cyst removal"*)	17
	#20	ALL (*"endometrioma removal"*)	10
	#21	ALL (*"endometriotic cystectomy"*)	30
	#22	ALL (*"laparoscopic cystectomy"*)	1552
	#23	ALL ("laparoscopic ovarian cystectomy")	1107
	#24	#17 OR #18 OR #19 OR #20 OR #21 OR #22 OR #23	3041
P	#25	(#6 AND #16) OR #24	6878
Interventions	#26	ALL (*hormonal)*	607887
and	#27	ALL (*"oral contraceptives")*	113903
Comparators	#28	ALL (*"oral contraception")*	21082
	#29	ALL ("combined pills")	439
	#30	ALL (estroprogestin)	181
	#31	ALL (progestin)	87591
	#32	ALL (dienogest)	2572
	#33	ALL ("depot medroxyprogesterone acetate")	4494
	#34	ALL ("Depo-provera")	4125
	#35	ALL ("LNG-IUS")	1534
	#36	ALL (Mirena)	2878
	#37	ALL ("levonorgestrel intrauterine system")	2261
	#38	ALL ("levonorgestrel-releasing intrauterine system")	4269
	#39	ALL ("gonadotropin releasing hormone agonist")	19825
	#40	ALL ("GnRH agonist")	15746
	#41	ALL ("GnRH analogue")	11459
	#42	ALL (expectant)	25050
I and C	#43	#26 OR #27 OR #28 OR #29 OR #30 OR #31 OR #32 OR #33 OR #34 OR #35 OR #36 OR #37 OR #38 OR #39 OR #40 OR #41 OR #42	778567
Outcomes	#44	ALL (recurrence)	1072479
	#45	ALL (dysmenorrhoea)	5066
	#46	ALL (dysmenorrhea)	18026
	#47	ALL ("painful menstruation")	222
	#48	ALL ("menstrual pain")	1956
	#49	#45 OR #46 OR #47 OR #48	20610
	#50	ALL ("pelvic pain")	31800
O	#51	#44 OR #49 OR #50	1116092
P, I/C &O	#52	#25 AND #43 AND # 51	1689
