# Supplementary material for: Postoperative hormonal treatment for prevention of endometrioma recurrence after ovarian cystectomy: a systematic review and network meta‐analysis
Source: BJOG. 2020 Jul 14;128(1):25–35. doi: 10.1111/1471-0528.16366 (PMC7754428; doi:10.1111/1471-0528.16366)
Supplement: Supplementary file 2 — Figure S1. Risk of bias assessments for RCTs. Figure S2. Forest plot of pairwise meta‐analysis of endometrioma recurrence based on RCT network. Figure S3. Network map of RCTs comparing hormonal treatments for prevention of endometrioma recurrence. Figure S4. Network contribution plot of RCTs for endometrioma recurrence outcome. Figure S5. Rankograms for hormonal network of RCTs showing the probability for each regimen being at a particular order in lowering endometrioma recurrence. Figure S6. Comparison‐adjusted funnel plot for network meta‐analysis of RCTs on endometrioma recurrence outcome. Figure S7. Forest plot of pairwise meta‐analysis of endometrioma recurrence based on cohort network. Figure S8. Funnel plot of cyclic OC versus expectant management and GnRHa versus expectant management for endometrioma recurrence based on cohort data. Figure S9. Network map of cohorts comparing among hormonal treatments for prevention of endometrioma recurrence. Figure S10. Network contribution plot of cohorts for endometrioma recurrence outcome. Figure S11. Rankograms for hormonal network of cohorts showing the probability for each regimen being at a particular order in lowering endometrioma recurrence. Figure S12. Comparison‐adjusted funnel plot for network meta‐analysis of cohorts on endometrioma recurrence outcome. [file BJO-128-25-s001.pdf]

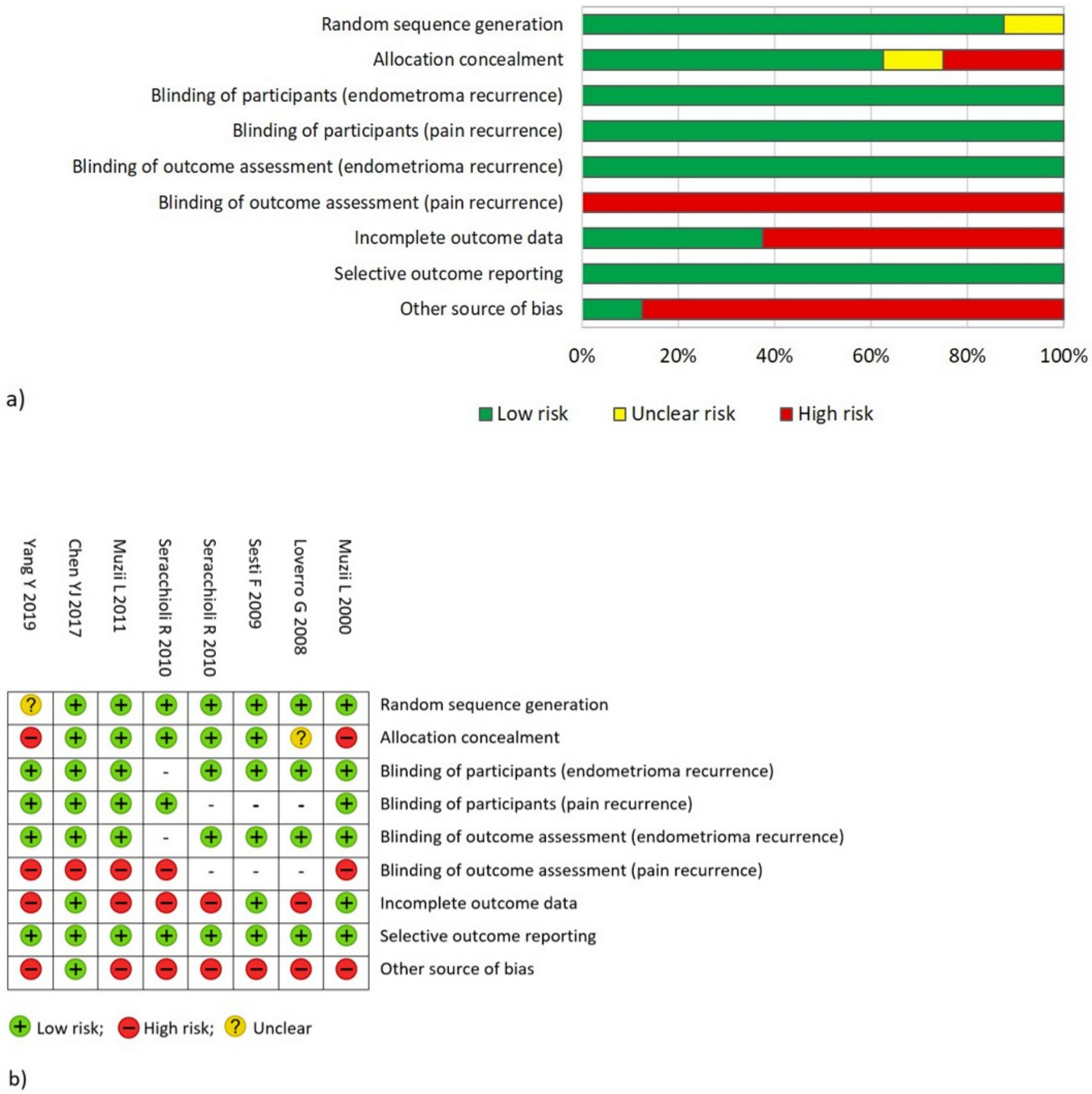

Figure S1. Risk of bias assessments for RCTs; a) Summary by domain b) Risk of bias for each included study and domain.

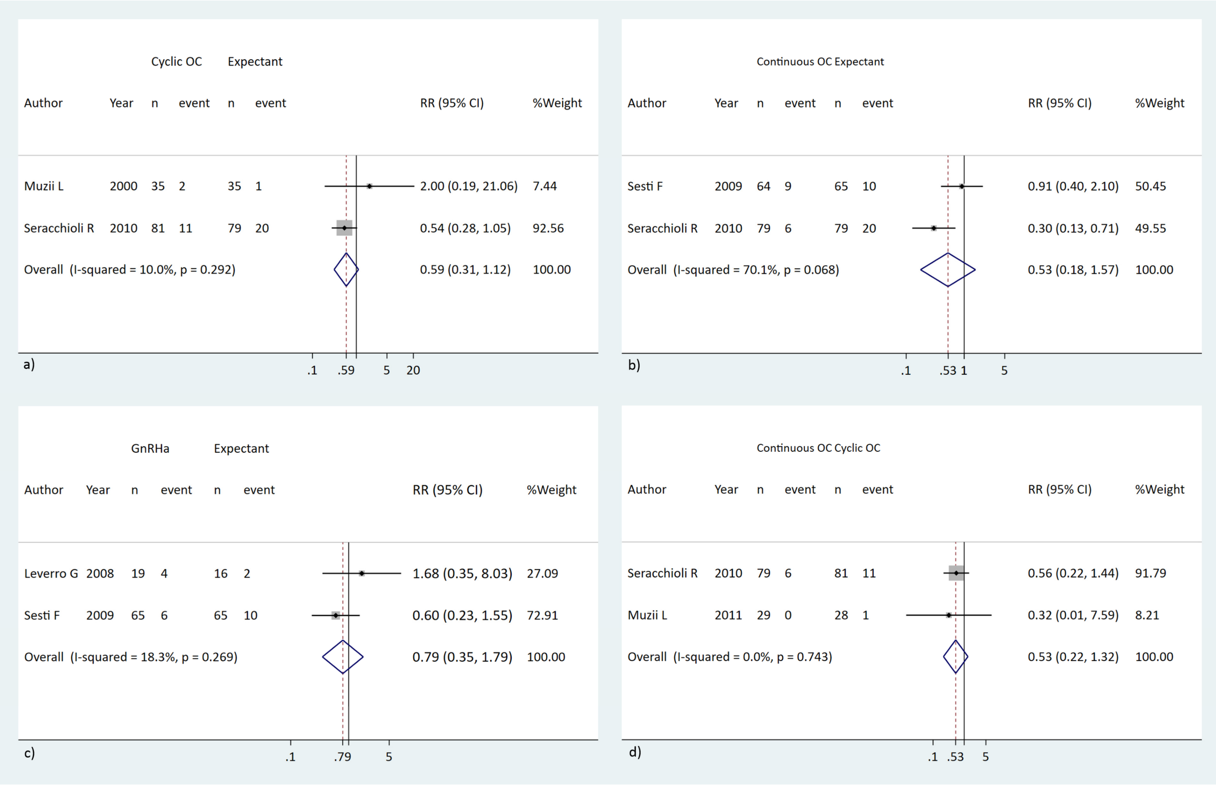

Figure S2. Forest plot of pairwise meta-analysis of endometrioma recurrence based on RCT network; a) Cyclic OC vs. expectant, b) Continuous OC vs. expectant, c) GnRHa vs. expectant, d) Continuous OC vs. cyclic OC.

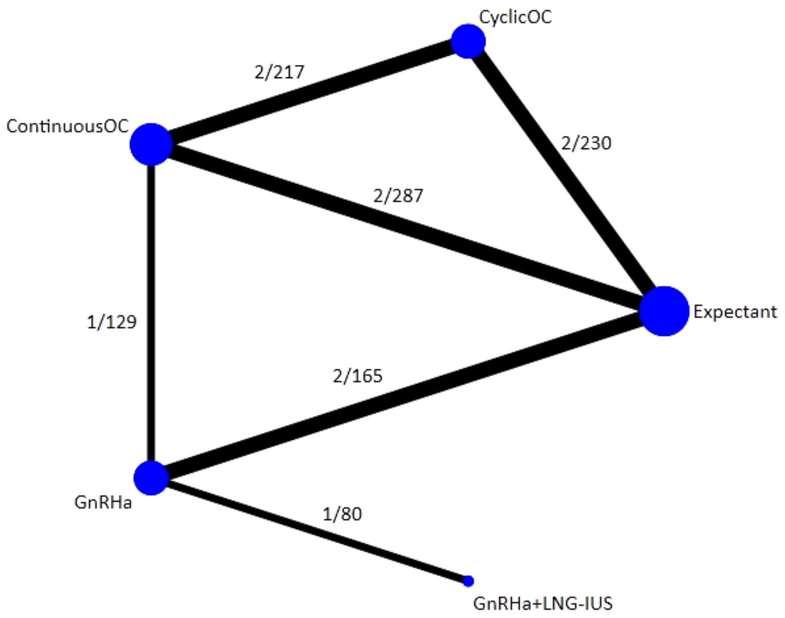

Figure S3. Network map of RCTs comparing among hormonal treatments for prevention of endometrioma recurrence; Numbers above edge are number of studies and included subjects in the corresponding comparison.

	Direct comparisons in the network					
	AvsB	AvsC	AvsD	BvsC	CvsD	DvsE
**Network meta-analysis estimates**						
Mixed estimates						
AvsB	48.4	13.0	8.5	21.6	8.5	·
AvsC	20.4	25.7	16.8	20.4	16.8	·
AvsD	10.3	13.1	42.9	10.3	23.4	·
BvsC	24.3	14.7	9.6	41.8	9.6	·
CvsD	10.8	13.7	24.6	10.8	40.0	
DvsE	·	·	·	·	·	100.0
Indirect estimates						
AvsE	6.2	7.9	25.8	6.2	14.1	39.9
BvsD	26.7	0.5	26.1	23.1	23.6	·
BvsE	17.8	0.3	17.5	15.4	15.8	33.2
CvsE	6.6	8.3	14.9	6.6	24.3	39.2
**Entire network**	16.6	8.9	19.5	15.2	18.4	21.4
**Included studies**	2	2	2	2	1	1

Figure S4. Network contribution plot of RCTs for endometrioma recurrence outcome; Values are percentages; A, expectant; B, Cyclic OC; C, Continuous OC; D, GnRHa; E, GnRHa+LNG-IUS.

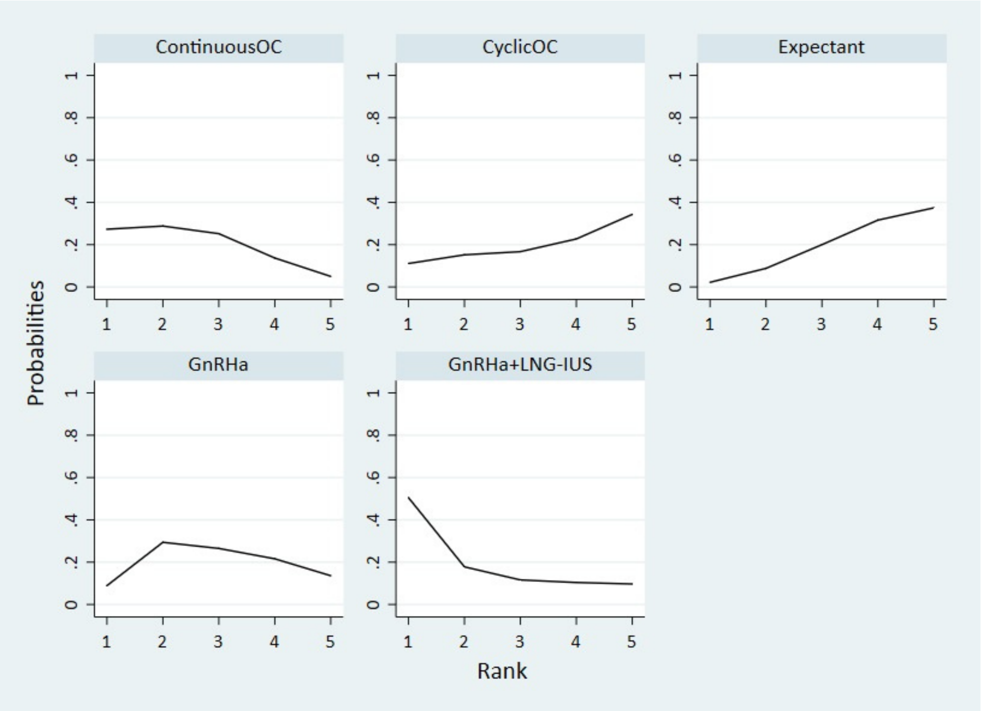

Figure S5. Rankograms for hormonal network of RCTs showing the probability for each regimen being at a particular order in lowering endometrioma recurrence.

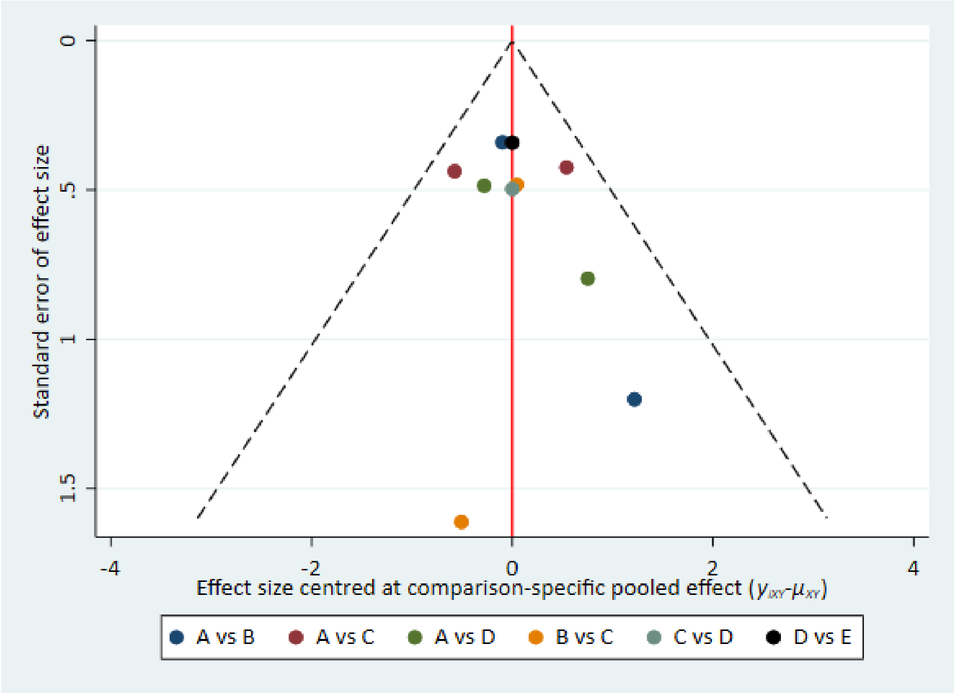

Figure S6. Comparison-adjusted funnel plot for network meta-analysis of RCTs on endometrioma recurrence outcome; A, expectant; B, Cyclic OC; C, Continuous OC; D, GnRHa; E, GnRHa+LNG-IUS.

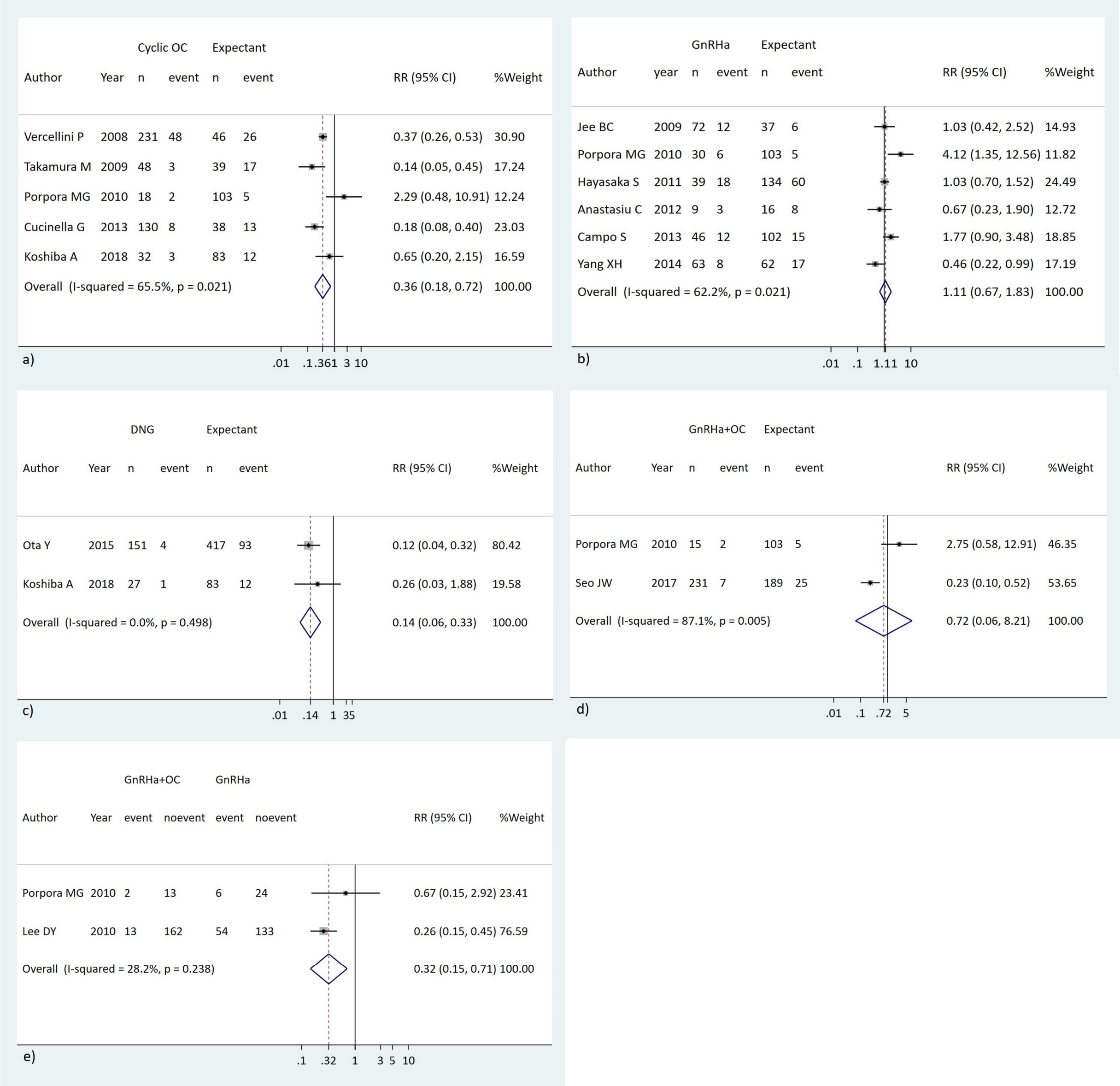

Figure S7. Forest plot of pairwise meta-analysis of endometrioma recurrence based on cohort network; a) Cyclic OC vs. expectant, b) GnRHa vs. expectant, c) DNG vs. expectant, d) GnRHa+OC vs. expectant, e) GnRHa+OC vs. GnRHa.

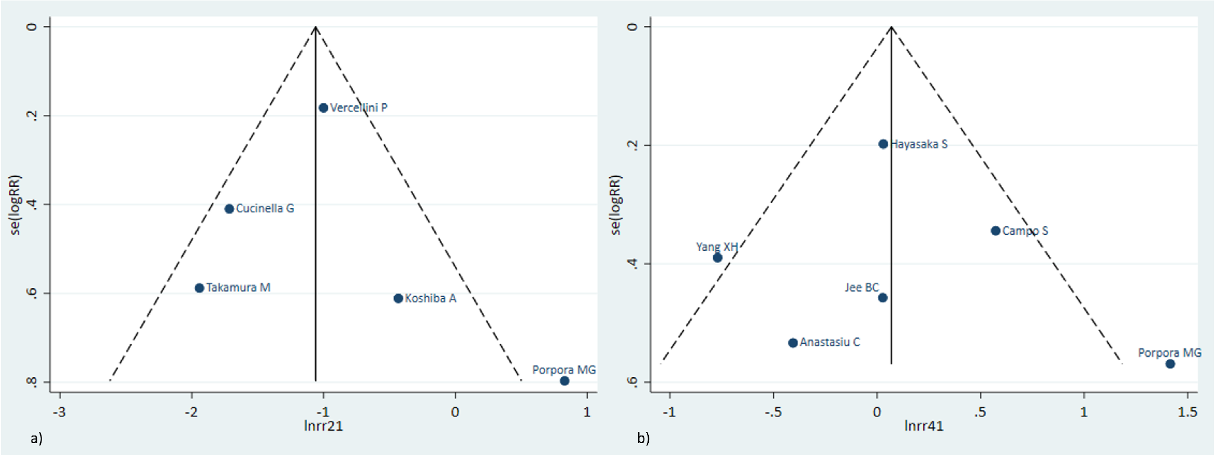

Figure S8. Funnel plot of cyclic OC vs. expectant (a); and GnRHa vs. expectant (b) for endometrioma recurrence based on cohort data.

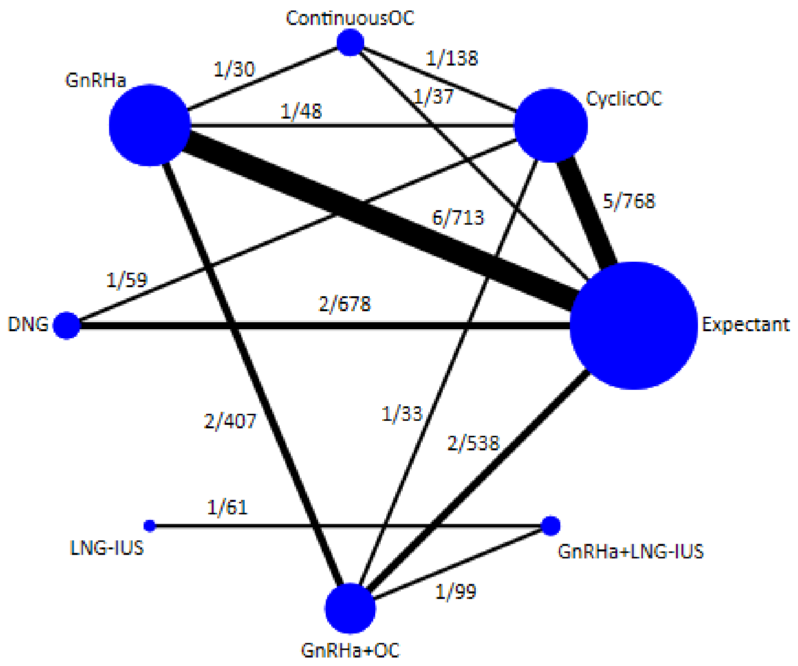

Figure S9. Network map of cohorts comparing among hormonal treatments for prevention of endometrioma recurrence; Numbers above edge are number of studies and included subjects in the corresponding comparison.

Direct comparisons in the network

Network meta-analysis estimates	AvsB	AvsC	AvsD	AvsE	AvsG	BvsC	BvsD	BvsE	BvsG	CvsD	DvsG	FvsH	GvsH
**Mixed estimates**													
AvsB	67.2	[illegible]	7.2	0.3	0.3	3.6	0.1	0.3	-	7.5	0.3	-	13.1
AvsC	0.2	51.6	[illegible]	4.0	1.0	8.5	8.6	4.0	4.9	0.4	4.0	-	-
AvsD	3.9	4.5	64.0	[illegible]	2.2	0.1	6.2	0.4	3.1	9.2	5.3	-	0.8
AvsE	0.5	4.3	1.2	74.5	0.1	3.7	1.6	11.1	0.9	1.2	0.8	-	0.1
AvsG	2.1	4.8	30.9	0.6	5.3	[illegible]	1.9	0.6	8.3	5.1	37.8	-	0.4
BvsC	5.4	8.1	0.4	3.2	0.4	48.2	8.3	3.2	4.7	12.9	4.2	-	1.0
BvsD	0.2	16.6	34.9	2.9	0.7	16.9	11.3	2.9	6.1	0.5	6.9	-	-
BvsE	1.7	15.2	4.4	39.5	0.5	13.2	5.7	9.2	3.3	4.2	2.8	-	0.3
BvsG	-	11.7	21.5	2.0	4.0	11.7	7.6	2.0	10.4	-	29.0	-	-
CvsD	17.0	0.6	38.8	1.6	1.5	19.7	0.4	1.6	-	14.0	[illegible]	[illegible]	3.3
DvsG	0.3	3.1	11.9	0.6	6.0	3.5	2.8	0.6	9.5	0.8	61.0	[illegible]	0.1
FvsH	[illegible]	-	-	-	-	-	-	-	-	-	[illegible]	100.0	-
GvsH	67.2	0.1	7.2	0.3	0.3	3.6	0.1	0.3	-	7.5	0.3	[illegible]	13.1
**Indirect estimates**													
AvsF	[illegible]	0.1	7.2	0.3	0.3	3.6	0.1	0.3	-	7.5	0.3	-	13.1
AvsH	[illegible]	0.1	7.2	0.3	0.3	3.6	0.1	0.3	-	7.5	0.3	-	13.1
BvsF	[illegible]	0.1	7.2	0.3	0.3	3.6	0.1	0.3	-	7.5	0.3	-	13.1
BvsH	67.2	0.1	7.2	0.3	0.3	3.6	0.1	0.3	-	7.5	0.3	-	13.1
CvsE	[illegible]	0.6	1.4	40.4	0.2	18.4	3.8	4.8	2.2	7.3	2.0	-	3.1
CvsF	67.2	0.1	7.2	0.3	0.3	3.6	0.1	0.3	-	7.5	0.3	-	13.1
CvsG	[illegible]	1.1	20.4	1.3	3.9	15.0	1.1	1.3	4.6	9.0	28.4	-	2.3
CvsH	67.2	0.1	7.2	0.3	0.3	3.6	0.1	0.3	-	7.5	0.3	-	13.1
DvsE	[illegible]	0.1	34.2	40.0	1.1	2.1	4.3	6.2	2.2	4.4	3.3	-	0.4
DvsF	[illegible]	0.1	7.2	0.3	0.3	3.6	0.1	0.3	-	7.5	0.3	-	13.1
DvsH	[illegible]	0.1	7.2	0.3	0.3	3.6	0.1	0.3	-	7.5	0.3	-	13.1
EvsF	67.2	0.1	7.2	0.3	0.3	3.6	0.1	0.3	-	7.5	0.3	-	13.1
EvsG	[illegible]	1.5	20.8	30.5	3.6	-	2.0	5.0	6.1	3.0	25.8	-	0.2
EvsH	[illegible]	0.1	7.2	0.3	0.3	3.6	0.1	0.3	-	7.5	0.3	-	13.1
FvsG	67.2	0.1	7.2	0.3	0.3	3.6	0.1	0.3	-	7.5	0.3	-	13.1
**Entire network**	27.0	5.2	15.5	10.7	1.5	8.5	2.8	2.4	3.0	5.9	9.9	2.3	5.3
**Included studies**	5	1	6	2	2	1	1	1	1	1	2	1	1

Figure S10. Network contribution plot of cohorts for endometrioma recurrence outcome; Values are percentages; A, expectant; B, Cyclic OC; C, Continuous OC; D, GnRHa; E, DNG; F, LNG-IUS; G, GnRHa+OC; H, GnRHa+LNG-IUS.

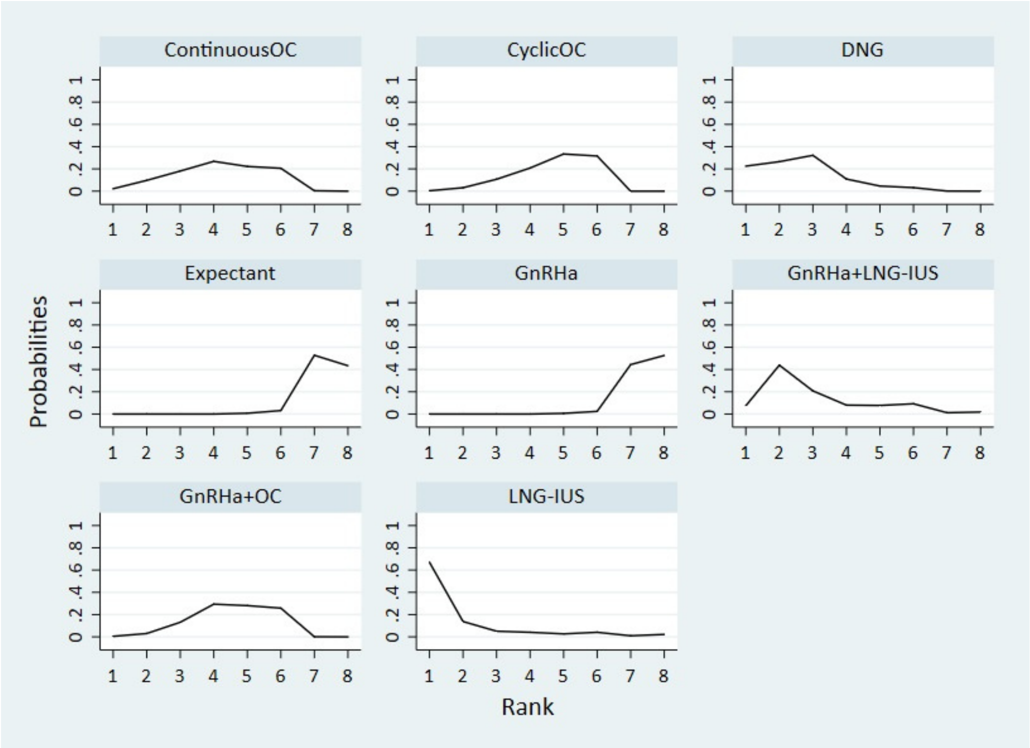

Figure S11. Rankograms for hormonal network of cohorts showing the probability for each regimen being at a particular order in lowering endometrioma recurrence.

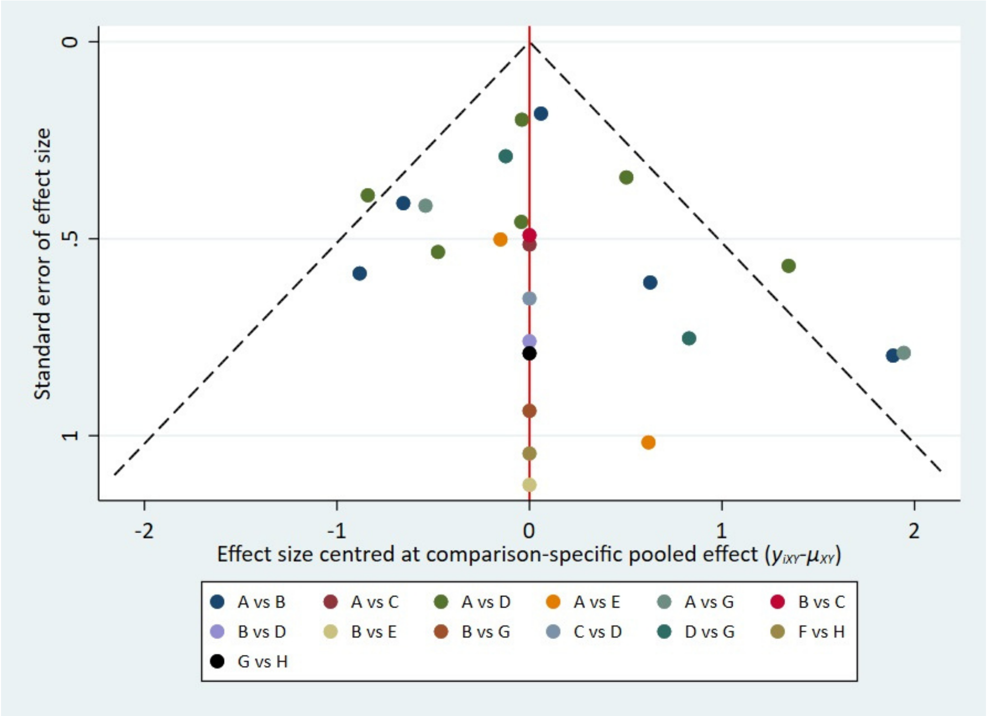

Figure S12. Comparison-adjusted funnel plot for the network meta-analysis of cohorts for endometrioma recurrence outcome; A, expectant; B, Cyclic OC; C, Continuous OC; D, GnRHa; E, DNG; F, LNG-US; G, GnRHa+OC; H, GnRHa+LNG-IUS.
