# Supplementary material for: Postoperative hormonal treatment for prevention of endometrioma recurrence after ovarian cystectomy: a systematic review and network meta‐analysis
Source: BJOG. 2020 Jul 14;128(1):25–35. doi: 10.1111/1471-0528.16366 (PMC7754428; doi:10.1111/1471-0528.16366)
Supplement: Supplementary file 3 — Table S1. Characteristics of included cohorts. Table S2. Risk of bias assessment of included cohort studies. Table S3. Multiple treatment comparison of RCTs network for endometrioma recurrence outcome: a sensitivity analysis by excluding two studies with continued hormonal treatments Table S4. Distribution of effect modifiers between each pairwise comparison across network of RCTs for endometrioma recurrence. Table S5. Explore sources of heterogeneity: meta‐regression Table S6. Distribution of effect modifiers between each pairwise comparison across network of cohorts for endometrioma recurrence. Table S7. Multiple treatment comparison of cohort network for endometrioma recurrence outcome in a subgroup continued hormonal treatment group. Table S8. Multiple treatment comparison of cohort network for endometrioma recurrence outcome in a subgroup continued hormonal treatment group with removing LNG‐IUS. Table S9. Multiple treatment comparison of cohort network for endometrioma recurrence outcome in a subgroup with discontinued treatment up to last follow up. Table S10. Relative effect estimates of treatment comparisons of RCT and cohort network for endometrioma recurrence. Table S11. Characteristics of subjects and RRs (95% CI) in GnRHa versus expectant management comparison; RCT and cohort study. Table S12. Characteristics of subjects in expectant management, cyclic OC and GnRHa intervention among RCTs and cohort studies. Table S13. Estimation of treatment effects on recurrence adjusting for study designs: a multilevel, mixed‐effect logit model. [file BJO-128-25-s002.pdf]

Table S1. Characteristics of included cohorts

Author, Year	Country	Included participants	Mean age (yr.)	Interventions	Duration of treatment (mo.)	N	Definition of outcomes		Follow up (mo.)
							Endometrioma recurrence	Pain recurrence	
Zhu S[29], 2018	China	Endometrioma stage 3&4	29.8	1. GnRHa<br>2. Expectant	24	96<br>67	Not defined	Not assessed	48
Koshiba A[37], 2018	Japan	Endometrioma underwent surgery	33.0	1. DNG<br>2. Cyclic OC<br>3. Expectant	40<br>21<br>-	27<br>32<br>83	Cyst > 2 cm	Not assessed	38
Seo JW[38], 2017	Korea	Endometrioma stage 3&4	30.9	1. GnRHa plus OC<br>2. Expectant	Entire FU<br>-	231<br>189	Cyst ≥ 2 cm	Not assessed	29
Lee DY[39], 2016	Korea	Symptomatic endometrioma stage 3&4	29.7	1. GnRHa plus OC<br>2. DNG	6<br>6	28<br>36	Not assessed	Not defined	6
Kim ML[40], 2016	Korea	Endometrioma stage 3&4	36.2	1. GnRHa plus LNG-IUS<br>2. LNG-IUS	Entire FU<br>Entire FU	41<br>20	Cyst ≥ 2 cm	Not assessed	> 6 (43)
Ota Y[41], 2015	Japan	Endometrioma stage 3&4	33.0	1. DNG<br>2. Expectant	60<br>-	151<br>417	Cyst ≥ 2 cm	Not assessed	60
Yang XH[42], 2014	China	Endometrioma underwent surgery	N.A.	1. GnRHa<br>2. Expectant	3<br>-	63<br>62	Not defined	Not assessed	18
Campo S[43], 2013	Sweden	Endometrioma underwent surgery	32.1	1. GnRHa<br>2. Expectant	6<br>-	46<br>102	Cyst ≥ 2 cm	Not assessed	30
Cucinella G[44], 2013	Italy	Endometrioma stage 3&4	29.0	1. Cyclic OC<br>2. Expectant	24<br>-	130<br>38	Not defined	Not defined	24
Cho S[45], 2013	Korea	Endometrioma stage 3&4	37.9	1. GnRHa plus LNG-IUS<br>2. GnRHa plus OC	Entire FU<br>Entire FU	42<br>57	Cyst ≥ 2 cm	Not assessed	24
Vlahos N[46], 2013	Greece	Endometriosis underwent surgery (endometrioma-sub cohort)	27.3	1. Continuous OC<br>2. Cyclic OC	21<br>23	54<br>84	Not defined	Not assessed	> 6 (21-23)

Anastasiu C[47], 2012	Romania	Endometrioma underwent surgery	N.A.	1. GnRHa<br>2. Continuous OC<br>3. Expectant	6<br>12<br>-	9<br>21<br>16	Not defined	Not assessed	12
Hayasaka S[48], 2011	Japan	Endometrioma underwent surgery	31.1	1. GnRHa<br>2. Expectant	3-6<br>-	18<br>60	Cyst ≥ 1.5 cm	Not assessed	12
Lee DY[49], 2010	Korea	Endometrioma stage 3&4	29.7	1. GnRHa plus OC<br>2. GnRHa	Entire FU<br>3-6	175<br>187	Cyst ≥ 2 cm	Not assessed	35
Porpora MG[50], 2010	Italy	Endometrioma stage 3&4	31.5	1. GnRHa plus OC<br>2. GnRHa<br>3. Cyclic OC<br>4. Expectant	12<br>3<br>12<br>-	15<br>30<br>18<br>103	Not defined	Not assessed	>36
Jee BC[18], 2009	Korea	Endometrioma stage 3&4	33.4	1. GnRHa<br>2. Expectant	3-6<br>-	72<br>37	Cyst > 1 cm	Not assessed	20
Takamura M[51], 2009	Japan	Endometrioma underwent surgery	33.4	1. Cyclic OC<br>2. Expectant	24<br>-	48<br>39	Cyst > 2 cm	Not assessed	24
Vercellini P[52], 2008	Italy	Endometrioma stage 3&4	30.8	1. Cyclic OC<br>2. Expectant	24<br>-	231<br>46	Cyst > 2 cm	Not assessed	24

N.A.: not available

Table S2. Risk of bias assessment of included cohort studies

Author, year	Bias due to confounding	Bias in selection of participants into the study	Bias in classification of interventions	Bias due to deviations from intended interventions	Bias due to missing data	Bias in measurement of outcomes	Bias in selection of the reported result	Overall RoB
Zhu S[29], 2018	Serious	Low	Low	NI	Low	NI	Low	**Serious**
Koshiba A[37], 2018	Serious	Serious	Low	Moderate	Moderate	Low	Low	**Serious**
Seo JW[38], 2017	Serious	Low	Low	Moderate	Moderate	Serious	Low	**Serious**
Lee DY[39], 2016	Moderate	Low	Low	Low	Moderate	Serious	Low	**Serious**
Kim ML[40], 2016	Moderate	Low	Low	Moderate	Low	Low	Low	**Moderate**
Ota Y[41], 2015	Serious	Low	Low	Moderate	Low	Low	Low	**Serious**
Yang XH[42], 2014	Critical	Low	Low	Low	Low	NI	Low	**Critical**
Campo S[43], 2013	Critical	Low	Low	Low	Low	Low	Low	**Critical**
Cucinella G[44], 2013	Serious	Low	Low	Moderate	Moderate	Low	Low	**Serious**
Cho S[45], 2013	Moderate	Low	Low	Low	Low	Low	Low	**Moderate**
Vlahos N[46], 2013	Serious	Low	Low	NI	Low	Low	Low	**Serious**
Anastasiu C[47], 2012	Critical	Low	Low	NI	NI	NI	Low	**Critical**
Hayasaka S[48], 2011	Critical	Low	Low	Low	Low	Low	Low	**Critical**
Lee DY[49], 2010	Moderate	Moderate	Low	Moderate	Low	Low	Low	**Moderate**
Porpora MG[50], 2010	Critical	Serious	Low	NI	NI	Low	Low	**Critical**
Jee BC[18], 2009	Serious	Low	Low	Low	Low	Low	Low	**Serious**
Takamura M[51], 2009	Moderate	Low	Low	Moderate	Low	Low	Low	**Moderate**
Vercellini P[52], 2008	Moderate	Low	Low	Moderate	Low	Low	Low	**Moderate**

NI: no information

Table S3. Multiple treatment comparison of RCTs network for endometrioma recurrence outcome: A sensitivity analysis by excluding two studies with continued hormonal treatments

Reference treatment	Risk ratio (95% CI)			
	**Expectant**	**Cyclic OC**	**Continuous OC**	**GnRHa**
**Expectant**	**52.1**	2.33 (0.35,15.74)	0.99 (0.45,2.20)	0.79 (0.35,1.77)
**Cyclic OC**	0.43 (0.06,2.89)	**17.5**	0.43 (0.06,3.01)	0.34 (0.04,2.58)
**Continuous OC**	1.01 (0.45,2.23)	2.35 (0.33,16.63)	**54.6**	0.79 (0.32,1.98)
**GnRHa**	1.27 (0.56,2.85)	2.96 (0.39,22.64)	1.26 (0.50,3.14)	**75.8**

Each off-diagonal cell contains RR (95% CI). Each diagonal cell contains SUCRA.

Table S4. Distribution of effect modifiers between each pairwise comparison across network of RCTs for endometrioma recurrence

Author, year	Characteristics								RR (95%CI)
	Mean age (yr.)	rASRM stage IV (%)	rASRM score	Cyst size (mm.)	Bilateral cyst (%)	Pelvic adhesion (%)	Duration of treatment (mo.)	Follow up (mo.)	
**Cyclic OC vs. expectant**									
Seracchioli R[33], 2010*	29.5	54.4	N.A.	49.4	13.4	100	24	24	0.54 (0.28, 1.05)
Muzii L[30], 2000	27.5	N.A.	44.8	52.0	35.7	100	6	36	2.00 (0.19, 21.06)
**Continuous OC vs. expectant**									
Seracchioli R[33], 2010*	29.5	54.4	N.A.	49.4	13.4	100	24	24	0.30 (0.13, 0.71)
Sesti F[32], 2009*	30.8	15.7	N.A.	33.6	17.9	N.A.	6	18	0.91 (0.39, 2.10)
**GnRHa vs. expectant**									
Sesti F[32], 2009*	30.8	15.7	N.A.	33.6	17.9	N.A.	6	18	0.60 (0.23, 1.55)
Loverro G[31], 2008	N.A.	N.A.	N.A.	N.A.	N.A.	N.A.	3	60	1.68 (0.35, 8.03)
**Continuous OC vs. cyclic OC**									
Muzii L[35], 2011	30.5	N.A.	41.3	50.5	N.A.	100	6	24	0.32 (0.01, 7.59)
Seracchioli R[33], 2010*	29.5	54.4	N.A.	49.4	13.4	100	24	24	0.56 (0.22, 1.44)
**GnRHa vs. continuous OC**									
Sesti F[32], 2009*	30.8	15.7	N.A.	33.6	17.9	N.A.	6	18	0.66 (0.25, 1.74)

N.A.: not available; *multi-arm trial

Table S5. Explore sources of heterogeneity: Meta-regression

	**RR (95% CI)**	**I^2**
**Cyclic OC vs. expectant**	0. 36 (0.18, 0.72)	65.47
Meta-regression		
Continued or discontinued treatment	0.25 (0.09, 0.69)	49.44
Age	1.10 (0.41, 2.96)	72.37
Cyst size	1.04 (0.70, 1.52)	70.39
Bilateral cyst	0.68 (0.08,5.66)	66.25
**GnRHa vs. expectant**	1.11 (0.67, 1.83)	62.19
Meta-regression		
Duration of follow up	0.87 (0.20, 3.79)	68.41

Table S6. Distribution of effect modifiers between each pairwise comparison across network of cohorts for endometrioma recurrence

Author, year	Characteristics								RR (95%CI)
	Mean age (yr.)	rASRM stage IV (%)	rASRM score	Cyst size (mm.)	Bilateral cyst (%)	Pelvic adhesion (%)	Duration of treatment (mo.)	Follow up (mo.)	
**Cyclic OC vs. expectant**									
Vercellini P[52], 2008	30.8	31.0	N.A.	54.1	27.1	N.A.	24	24	0.37 (0.26, 0.53)
Takamura M[51], 2009	33.4	N.A.	59.0	53.0	36.8	N.A.	24	24	0.14 (0.05, 0.45)
Porpora MG[50], 2010*	31.5	43.4	N.A.	49.0	31.3	N.A.	12	>36	2.29 (0.48, 10.91)
Cucinella G[44], 2013	29.0	54.8	N.A.	47.4	36.9	90	24	24	0.18 (0.08, 0.40)
Kosiba A[37], 2018	33.0	N.A.	N.A.	59.5	35.2	N.A.	21	38	0.65 (0.19, 2.15)
**Continuous OC vs. expectant**									
Anastasiu C[47], 2012	N.A.	N.A.	N.A.	N.A.	32.6	N.A.	12	12	0.38 (0.14, 1.05)
**GnRHa vs. expectant**									
Jee BC[18], 2009	33.4	38.5	N.A.	55.7	14.7	N.A.	3-6	20	1.03 (0.42, 2.52)
Porpora MG[50], 2010*	31.5	43.4.	N.A.	49.0	31.3	N.A.	3	>36	4.12 (1.35, 12.56)
Hayasaka S[48], 2011	31.1	N.A.	44.8	56.7	N.A.	N.A.	3-6	12	1.03 (0.70, 1.52)
Anastasiu C[47], 2012*	N.A.	N.A.	N.A.	N.A.	32.6	N.A.	6	12	0.67 (0.23, 1.89)
Campo S[43], 2013	32.1	N.A.	N.A.	48.8	20.9	N.A.	6	30	1.77 (0.90, 3.48)
Yang XH[42], 2014	N.A.	14.4	N.A.	N.A.	N.A.	N.A.	3	18	0.46 (0.22, 0.99)
**DNG vs. expectant**									
Ota Y[41], 2015	33.0	72.3	N.A.	N.A.	45.4	59.8	60	60	0.12 (0.04, 0.32)
Kosiba A[37], 2018*	33.0	N.A.	N.A.	59.5	35.2	N.A.	40	38	0.26 (0.04, 1.88)
**GnRHa+OC vs. expectant**									
Porpora MG[50], 2010*	31.5	43.4.	N.A.	49.0	31.3	N.A.	12	>36	2.75 (0.58, 12.91)
Seo JW[38], 2017	30.9	38.6	N.A.	N.A.	35.9	N.A.	29	29	0.23 (0.10, 0.52)
**Continuous OC vs. cyclic OC**									
Vlahos N[46], 2013	27.3	N.A.	N.A.	46.2	25.4	N.A.	23/21	>6	0.56 (0.21, 1.45)

**GnRHa vs. cyclic OC**									
Porpora MG[50], 2010*	31.5	43.4.	N.A.	49.0	31.3	N.A.	3/12	>36	1.80 (0.41, 7.98)
**DNG vs. cyclic OC**									
Kosiba A[37], 2018*	33.0	N.A.	N.A.	59.5	35.2	N.A.	40/21	38	0.39 (0.04, 3.58)
**GnRHa+OC vs. cyclic OC**									
Porpora MG[50], 2010*	31.5	43.4.	N.A.	49.0	31.3	N.A.	12	>36	1.20 (0.19, 7.53)
**GnRHa vs. continuous OC**									
Anastasiu C[47], 2012*	N.A.	N.A.	N.A.	N.A.	32.6	N.A.	6/12	12	1.75 (0.49, 6.28)
**GnRHa+OC vs. GnRHa**									
Porpora MG[50], 2010*	31.5	43.4.	N.A.	49.0	31.3	N.A.	12/3	>36	0.67 (0.15, 2.92)
Lee DY[49], 2010	29.7	45.6	N.A.	59.1	35.9	N.A.	35/3-6	35	0.26 (0.15, 0.46)

N.A.: not available; *multi-arm trial

Table S7. Multiple treatment comparison of cohort network for endometrioma recurrence outcome in a subgroup of continued hormonal treatment group

Reference treatment	Risk ratio (95% CI)						
	Expectant	Cyclic OC	Continuous OC	DNG	LNG-IUS	GnRHa + OC	GnRHa + LNG-IUS
**Expectant**	**0.3**	0.30 (0.18,0.48)	0.25 (0.11,0.56)	0.14 (0.05,0.37)	0.04 (0.00,0.61)	0.23 (0.09,0.61)	0.10 (0.02,0.70)
**Cyclic OC**	3.37 (2.07,5.48)	**33.2**	0.83 (0.36,1.90)	0.47 (0.16,1.41)	0.12 (0.01,2.14)	0.77 (0.26,2.29)	0.35 (0.05,2.49)
**Continuous OC**	4.05 (1.77,9.27)	1.20 (0.53,2.75)	**44.0**	0.57 (0.16,2.05)	0.14 (0.01,2.78)	0.93 (0.26,3.33)	0.42 (0.05,3.35)
**DNG**	7.16 (2.68,19.13)	2.13 (0.71,6.40)	1.77 (0.49,6.40)	**67.8**	0.25 (0.01,5.15)	1.64 (0.41,6.54)	0.74 (0.09,6.33)
**LNG-IUS**	28.23 (1.64,485.94)	8.39 (0.47,150.44)	6.97 (0.36,135.00)	3.94 (0.19,79.97)	**90.6**	6.47 (0.45,93.84)	2.93 (0.35,24.27)
**GnRHa + OC**	4.36 (1.65,11.53)	1.30 (0.44,3.84)	1.08 (0.30,3.86)	0.61 (0.15,2.43)	0.15 (0.01,2.24)	**44.2**	0.45 (0.09,2.33)
**GnRHa + LNG-IUS**	9.65 (1.44,64.73)	2.87 (0.40,20.44)	2.38 (0.30,18.98)	1.35 (0.16,11.47)	0.34 (0.04,2.83)	2.21 (0.43,11.36)	**70.0**

Each off-diagonal cell contains RR (95% CI). Each diagonal cell contains SUCRA.

Table S8. Multiple treatment comparison of cohort network for endometrioma recurrence outcome in a subgroup of continued hormonal treatment group with removing LNG-IUS

Reference treatment	Risk ratio (95% CI)				
	Expectant	Cyclic OC	Continuous OC	DNG	GnRHa + OC
**Expectant**	**0.0**	0.30 (0.18,0.48)	0.25 (0.11,0.56)	0.14 (0.05,0.37)	0.23 (0.09,0.61)
**Cyclic OC**	3.37 (2.07,5.48)	**42.8**	0.83 (0.36,1.90)	0.47 (0.16,1.41)	0.77(0.26,2.29)
**Continuous OC**	4.05 (1.77,9.27)	1.20 (0.53,2.75)	**58.3**	0.57 (0.16,2.05)	0.93 (0.26,3.32)
**DNG**	7.16 (2.68,19.13)	2.13 (0.71,6.40)	1.77 (0.49,6.40)	**87.2**	1.64 (0.41,6.53)
**GnRHa + OC**	4.37 (1.65,11.53)	1.30 (0.44,3.84)	1.08 (0.30,3.86)	0.61 (0.15,2.43)	**61.7**

Each off-diagonal cell contains RR (95% CI). Each diagonal cell contains SUCRA.

Table S9. Multiple treatment comparison of cohort network for endometrioma recurrence outcome in a subgroup discontinued treatment up to last follow-up

Reference treatment	Risk ratio (95% CI)			
	Expectant	Cyclic OC	GnRHa	GnRHa+OC
**Expectant**	**65.2**	1.18 (0.20,6.94)	1.21 (0.65,2.26)	1.41 (0.24,8.23)
**Cyclic OC**	0.85 (0.14,4.99)	**50.5**	1.03 (0.18,5.98)	1.20 (0.14,10.52)
**GnRHa**	0.83 (0.44,1.54)	0.97 (0.17,5.66)	**45.3**	1.17 (0.20,6.71)
**GnRHa+OC**	0.71 (0.12,4.11)	0.83 (0.10,7.30)	0.86 (0.15,4.92)	**39.0**

Each off-diagonal cell contains RR (95% CI). Each diagonal cell contains SUCRA.

Table S10. Relative effect estimates of treatment comparisons of RCT and cohort network for endometrioma recurrence

Treatment pair	RCT (6 studies)			Cohort (16 studies)		
	**No. direct comparison**	**Direct estimate (95% CI)**	**NMA estimate (95% CI)**	**No. direct comparison**	**Direct estimate (95% CI)**	**NMA estimate (95% CI)**
Cyclic OC vs. expectant	2	0.59 (0.31, 1.12)	0.90 (0.28,2.92)	5	0. 36 (0.18, 0.72)	0.35 (0.20,0.60)
Continuous OC vs. expectant	2	0.53 (0.18, 1.57)	0.59 (0.23,1.54)	1	0.38 (0.14, 1.05)	0.30 (0.11,0.77)
GnRHa vs. expectant	2	0.79 (0.35, 1.79)	0.72 (0.23,2.26)	6	1.11 (0.67, 1.83)	1.03 (0.65,1.62)
DNG vs. expectant	-	-	-	2	0.14 (0.06, 0.33)	0.14 (0.05,0.43)
LNG-IUS vs. expectant	-	-	-	-	-	0.05 (0.00,0.98)
GnRHa + OC vs. expectant	-	-	-	2	0.72 (0.06, 8.21)	0.33 (0.15,0.71)
GnRHa + LNG-IUS vs. expectant	-	-	0.48 (0.08,2.73)	-	-	0.15 (0.02,1.04)
Continuous OC vs. cyclic OC	2	0.53 (0.22, 1.32)	0.66 (0.19,2.25)	1	0.56 (0.21, 1.45)	0.85 (0.33,2.19)
GnRHa vs. cyclic OC	-	-	0.80 (0.18,3.62)	1	1.80 (0.41, 7.98)	2.93 (1.49,5.79)
DNG vs. cyclic OC	-	-	-	1	0.39 (0.04, 3.58)	0.40 (0.12,1.36)
LNG-IUS vs. cyclic OC	-	-	-	-	-	0.15 (0.01,2.91)
GnRHa + OC vs. cyclic OC	-	-	-	1	1.20 (0.19, 7.53)	0.95 (0.38,2.33)
GnRHa + LNG-IUS vs. cyclic OC	-	-	0.53 (0.07,3.94)	-	-	0.43 (0.06,3.15)
GnRHa vs. continuous OC	1	0.66 (0.25, 1.74)	1.21 (0.34,4.26)	1	1.75 (0.49, 6.28)	3.46 (1.26,9.49)
DNG vs. continuous OC	-	-	-	-	-	0.47 (0.11,2.02)
LNG-IUS vs. continuous OC	-	-	-	-	-	0.17 (0.01,3.77)
GnRHa + OC vs. continuous OC	-	-	-	-	-	1.11 (0.34,3.63)
GnRHa+ LNG-IUS vs. continuous OC	-	-	0.81 (0.13,4.97)	-	-	0.50 (0.06,4.27)
DNG vs. GnRHa	-	-	-	-	-	0.14 (0.04,0.46)
LNG-IUS vs. GnRHa	-	-	-	-	-	0.05 (0.00,0.95)
GnRHa + OC vs. GnRHa	-	-	-	2	0.32 (0.15, 0.71)	0.32 (0.15,0.69)
GnRHa+ LNG-IUS vs. GnRHa	1	0.67 (0.34, 1.30)	0.67 (0.18,2.48)	-	-	0.15 (0.02,1.01)
LNG-IUS vs. DNG	-	-	-	-	-	0.37 (0.02,8.60)
GnRHa + OC vs. DNG	-	-	-	-	-	2.37 (0.61,9.15)
GnRHa + LNG-IUS vs. DNG	-	-	-	-	-	1.07 (0.11,10.02)
GnRHa + OC vs. LNG-IUS	-	-	-	-	-	6.47 (0.37,111.99)
GnRHa + LNG-IUS vs. LNG-IUS	-	-	-	1	2.93 (0.38, 22.69)	2.93 (0.32,27.16)
GnRHa + LNG-IUS vs. GnRHa + OC	-	-	-	1	0.45 (0.09, 2.13)	0.45 (0.08,2.68)

Table S11. Characteristics of subjects and RRs (95%CI) in GnRHa vs. expectant comparison; RCTs and cohort studies

Characteristics	RCT			Cohorts						
	Loverro G[31], 2008	Sesti F[32], 2009	**Average**	Jee BC[18], 2009	Porpora MG[50], 2010	Hayasaka S[48], 2011	Anastasiu C[47], 2012	Campo S[43], 2013	Yang XH[42], 2014	**Average**
Mean age (yr.)	N. A.	30.8	30.8	33.4	31.5	31.1	N.A.	32.1	N.A.	31.9
rASRM scores	N.A.	N.A.	N.A.	N.A.	N.A.	44.8	N.A.	N.A.	N.A.	44.8
rASRM stage IV (%)	N.A.	15.7	15.7	38.5	43.4	N.A.	N.A.	N.A.	14.4	33.0
Cyst size (mm.)	N.A.	33.6	33.6	55.7	49.0	56.7	N.A.	48.8	N.A.	52.4
Bilateral cyst (%)	N.A.	17.9	17.9	14.7	31.3	N.A.	32.6	20.9	N.A.	24.3
Pelvic adhesion (%)	N.A.	N.A.	N.A.	N.A.	N.A.	N.A.	N.A.	N.A.	N.A.	N.A.
Duration of treatment (mo.)	3	6	-	3-6	3	3-6	6	3	6	-
Follow up (mo.)	60	18	-	20	>36	12	12	30	18	-
Definition of cyst recurrence	Not defined	> 2 cm	-	> 1 cm	Not defined	> 1.5 cm	Not defined	> 2 cm	Not defined	-
RR (95%CI)	1.68 (0.35, 8.03)	0.60 (0.23, 1.55)	0.79 (0.35, 1.79)	1.03 (0.42, 2.52)	4.12 (1.35, 12.56)	1.03 (0.70, 1.52)	0.67 (0.23, 1.89)	1.77 (0.90, 3.48)	0.46 (0.22, 0.99)	1.11 (0.67, 1.83)

Table S12. Characteristics of subjects in expectant, cyclic OC, and GnRHa intervention among RCTs and cohort studies

Author, year	Characteristics																	
	**Mean age (years)**			**Pelvic adhesion (%)**			**rASRM score**			**rASRM stage IV (%)**			**Size of cyst (mm.)**			**Bilateral cyst (%)**		
	Expectant	Cyclic OC	GnRHa	Expectant	Cyclic OC	GnRHa	Expectant	Cyclic OC	GnRHa	Expectant	Cyclic OC	GnRHa	Expectant	Cyclic OC	GnRHa	Expectant	Cyclic OC	GnRHa
**RCTs**																		
Chen YJ[36],2017	-	-	32.9	-	-	N.A.	-	-	50.4	-	-	60.0	-	-	57.8	-	-	37.5
Muzii L[35], 2011	-	30.6	-	-	100	-	-	40.4	-	-	N.A.	-	-	51.0	-	-	N.A.	-
Seracchioli R[33], 2010	30.1	29.7	-	100	100	-	N.A.	N.A.	-	56.5	50.7	-	48.0	49.0	-	14.5	10.7	-
Sesti F[32], 2009	31.3	-	30.8	N.A.	-	N.A.	N.A.	-	N.A.	15.0	-	17.2	35.8	-	30.8	15.0	-	20.7
Leverro G[31], 2008	N.A.	-	N.A.	N.A.	-	N.A.	N.A.	-	N.A.	N.A.	-	N.A.	N.A.	-	N.A.	N.A.	-	N.A.
Muzii L[30], 2000	26.8	28.2	-	100	100	-	46.1	43.4.		N.A.	N.A.		51.0	53.0	-	40.0	33.3	-
**Average**	**29.4**	**29.5**	**31.9**	**100**	**100**					**35.8**	**50.7**	**38.6**	**44.9**	**51.0**	**44.3**	**23.2**	**22.0**	**29.1**
**Cohort studies**																		
Koshiba A[37], 2018	32.0	29.0	-	N.A.	N.A.	-	N.A.	N.A.	-	N.A.	N.A.	-	54.0	71.0	-	36.1	31.3	-
Seo JW[38], 2017	33.5	-	-	N.A.	-	-	N.A.	-	-	37.0	-	-	59.2	-	-	21.7	-	-
Ota Y[41], 2015	33.2	-	-	50.1	-	-	N.A.	-	-	72.4	-	-	N.A.	-	-	44.6	-	-
Yang XH[42], 2014	N.A.	-	N.A.	N.A.	-	N.A.	N.A.	-	N.A.	N.A.	-	N.A.	N.A.	-	N.A.	N.A.	-	N.A.
Campo S[43], 2014	N.A.	-	N.A.	N.A.	-	N.A.	N.A.	-	N.A.	N.A.	-	N.A.	N.A.	-	N.A.	N.A.	-	N.A.
Cucinella G[44], 2013	30.4	29.4	-	N.A.	N.A.	--	N.A.	N.A.	-	57.9	53.8	-	41.0	46.2	-	34.2	37.7	-
Vlahos N[46], 2013	-	N.A.	-	-	N.A.	-	-	N.A.	-	-	N.A.	-	-	45.0	-	-	25.0	-
Anatasiu C[47], 2012	N.A.	N.A.	N.A.	N.A.	N.A.	N.A.	N.A.	N.A.	N.A.	N.A.	N.A.	N.A.	N.A.	N.A.	N.A.	12.5	33.3	66.7
Hayasaka S[48], 2011	N.A.	-	N.A.	N.A.	-	N.A.	N.A.	-	N.A.	N.A.	-	N.A.	N.A.	-	N.A.	N.A.	-	N.A.
Lee DY[49], 2010	-	-	30.1	-	-	N.A.	-	-	N.A.	-	-	46.5	-	-	61.0	-	-	36.4
Porpora MG[50], 2010	N.A.	N.A.	N.A.	N.A.	N.A.	N.A.	N.A.	N.A.	N.A.	N.A.	N.A.	N.A.	N.A.	N.A.	N.A.	N.A.	N.A.	N.A.
Jee BC[18], 2009	35.5	-	32.3	N.A.	-	N.A.	N.A.	-	N.A.	24.3	-	45.8	53.0	-	52.6	10.8	-	16.7
Takamura, M[51], 2009	N.A.	N.A.	-	N.A.	N.A.	-	N.A.	N.A.	-	N.A.	N.A.	-	N.A.	N.A.	-	N.A.	N.A.	-
Vercellini P[52], 2008	33.0	30.4	-	N.A.	N.A.	-	N.A.	N.A.	-	34.8	30.3	-	52.0	54.6	-	39.1	24.7	-
**Average**	**32.9**	**29.6**	**31.2**							**45.3**	**42.1**	**46.2**	**51.8**	**54.3**	**56.8**	**28.4**	**31.8**	**39.9**

N.A., not available

Table S13. Estimation of treatment effects on recurrence adjusting for study designs: A multilevel mixed-effect logit model

Treatment pair	RR (95% CI)
Cyclic OC vs. expectant	0.39 (0.28,0.54)
Continuous OC vs. expectant	0.40 (0.22,0.58)
GnRHa vs. expectant	0.98 (0.79,1.18)
DNG vs. expectant	0.14 (0.03,0.29)
LNG-IUS vs. expectant	0.18 (0.06,0.41)
GnRHa + OC vs. expectant	0.32 (0.21,0.50)
GnRHa + LNG-IUS vs. expectant	0.49 (0.29,0.85)
Continuous OC vs. cyclic OC	1.02 (0.59,1.56)
GnRHa vs. cyclic OC	2.48 (1.77,3.77)
DNG vs. cyclic OC	0.37 (0.09,0.89)
LNG-IUS vs. cyclic OC	0.48 (0.14,1.16)
GnRHa + OC vs. cyclic OC	0.81 (0.45,1.43)
GnRHa + LNG-IUS vs. cyclic OC	1.26 (0.67,2.29)
GnRHa vs. continuous OC	2.44 (1.57,4.25)
DNG vs. continuous OC	0.36 (0.09,0.83)
LNG-IUS vs. continuous OC	0.47 (0.11,1.18)
GnRHa + OC vs. continuous OC	0.79 (0.44,1.59)
GnRHa+ LNG-IUS vs. continuous OC	1.24 (0.63,2.49)
DNG vs. GnRHa	0.15 (0.04,0.32)
LNG-IUS vs. GnRHa	0.19 (0.06,0.46)
GnRHa + OC vs. GnRHa	0.33 (0.21,0.48)
GnRHa+ LNG-IUS vs. GnRHa	0.51 (0.29,0.87)
LNG-IUS vs. DNG	1.30 (0.25,3.67)
GnRHa + OC vs. DNG	2.20 (0.95,8.94)
GnRHa + LNG-IUS vs. DNG	3.44 (1.29,15.25)
GnRHa + OC vs. LNG-IUS	1.69 (0.74,5.99)
GnRHa + LNG-IUS vs. LNG-IUS	2.64 (1.15,7.65)
GnRHa + LNG-IUS vs. GnRHa + OC	1.56 (0.78,3.09)
